# The aging of ER-mitochondria communication: A journey from undifferentiated to aged cells

**DOI:** 10.3389/fcell.2022.946678

**Published:** 2022-08-19

**Authors:** Pablo Morgado-Cáceres, Gianella Liabeuf, Ximena Calle, Lautaro Briones, Jaime A. Riquelme, Roberto Bravo-Sagua, Valentina Parra

**Affiliations:** ^1^ Advanced Center of Chronic Diseases (ACCDiS), Facultad de Ciencias Químicas y Farmacéuticas e Instituto de Nutrición y Tecnología de los Alimentos (INTA), Universidad de Chile, Santiago, Chile; ^2^ Departamento de Bioquímica y Biología Molecular y Departamento de Química Farmacológica y Toxicológica, Facultad de Ciencias Químicas y Farmacéuticas, Universidad de Chile, Santiago, Chile; ^3^ Center for Integrative Biology, Faculty of Sciences, Universidad Mayor, Santiago, Chile; ^4^ Laboratorio de Obesidad y Metabolismo Energético (OMEGA), Instituto de Nutrición y Tecnología de los Alimentos (INTA), Universidad de Chile, Santiago, Chile; ^5^ Facultad de Salud y Ciencias Sociales, Escuela de Nutrición y Dietética, Universidad de las Américas, Santiago, Chile; ^6^ Departamento de Nutrición y Salud Pública, Facultad de Ciencias de la Salud y de los Alimentos, Universidad del Bío-Bío, Chillán, Chile; ^7^ Red de Investigación en Envejecimiento Saludable, Consorcio de Universidades del Estado de Chile, Santiago, Chile; ^8^ Red para el Estudio de Enfermedades Cardiopulmonares de alta letalidad (REECPAL), Universidad de Chile, Santiago, Chile

**Keywords:** endoplasmic reticulum, mitochondria, aging, cellular diffentiation, chronic diseases

## Abstract

The complex physiology of eukaryotic cells requires that a variety of subcellular organelles perform unique tasks, even though they form highly dynamic communication networks. In the case of the endoplasmic reticulum (ER) and mitochondria, their functional coupling relies on the physical interaction between their membranes, mediated by domains known as mitochondria-ER contacts (MERCs). MERCs act as shuttles for calcium and lipid transfer between organelles, and for the nucleation of other subcellular processes. Of note, mounting evidence shows that they are heterogeneous structures, which display divergent behaviors depending on the cell type. Furthermore, MERCs are plastic structures that remodel according to intra- and extracellular cues, thereby adjusting the function of both organelles to the cellular needs. In consonance with this notion, the malfunction of MERCs reportedly contributes to the development of several age-related disorders. Here, we integrate current literature to describe how MERCs change, starting from undifferentiated cells, and their transit through specialization, malignant transformation (i.e., dedifferentiation), and aging/senescence. Along this journey, we will review the function of MERCs and their relevance for pivotal cell types, such as stem and cancer cells, cardiac, skeletal, and smooth myocytes, neurons, leukocytes, and hepatocytes, which intervene in the progression of chronic diseases related to age.

## Introduction

Eukaryotic cells are composed of several organelle networks that are in charge of unique functions to coordinate complex intracellular processes. Therefore, intercommunication between organelle networks is important for proper cell function (López-Crisosto et al., 2015; Aoyama-Ishiwatari and Hirabayashi, 2021). Accordingly, mitochondria maintain communication with various other organelles, such as the endoplasmic reticulum (ER), Golgi apparatus, lysosomes, and peroxisomes, and even establish physical contacts with each one of them (Liao et al., 2020; Silva et al., 2020). So far, many studies have compiled evidence about ER-mitochondria interactions, making it one of the most studied inter-organelle communication systems (López-Crisosto et al., 2015; Bravo-Sagua et al., 2016; López-Crisosto et al., 2017; Lopez-Crisosto et al., 2021).

**FIGURE 1:**
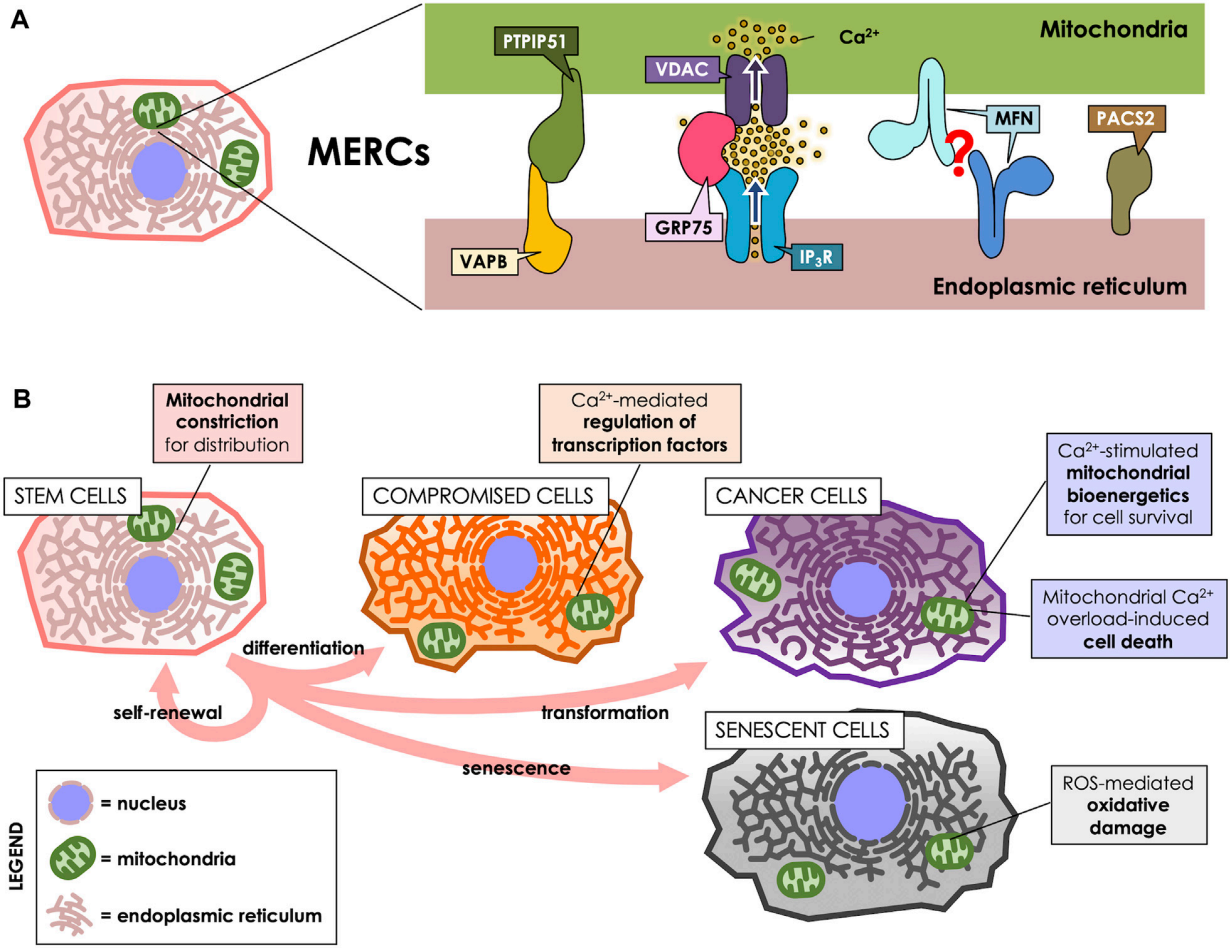
MERCs participate throughout the cell life cycle. **(A)** MERCs are sites of close apposition between ER and mitochondria, maintained by proteins such as Vesicle-associated membrane protein-associated protein B (VAPB) and Protein tyrosine phosphatase-interacting protein 51 (PTPIP51), which form a bridge between the surfaces of both organelles. GRP75, on the other hand, tethers the IP_3_R and VDAC Ca^2+^ channels to allow for an efficient Ca^2+^ transfer. Mitofusins (MFNs) are present at the surface of both ER and mitochondria, purportedly forming a tether between both; however, this is a matter of controversy, as they have been proposed to also antagonize ER-mitochondria interaction. PACS-2, instead, is an ER-resident sorting protein enriched in MERCs, which governs MERCs composition. **(B)** MERCs have different functions according to the cell’s requirements. They participate in cell division, differentiation, death, survival and senescence via regulation of mitochondrial dynamics, Ca^2+^ homeostasis or ROS production.

Mitochondria-ER contacts (MERCs) are defined as the sites of physical proximity between the surfaces of both organelles (<100 nm) without fusion of their membranes (Csordás et al., 2018). Because of their short span, the gold standard for MERCs detection is electron microscopy (Csordás et al., 2018). Additionally, and due to their presence in a broad variety of cell types, they are seemingly prevalent in all cells containing mitochondria and ER. At the molecular level, MERCs structure is maintained by proteins that bridge the surfaces of ER and mitochondria (Figure 1A), which can be characterized through cellular fractionation (Vance, 2014). ER fractions that are tethered to mitochondria–and thus co-purify with the mitochondrial fraction- are termed mitochondria-associated membranes (MAMs). Therefore, from now on we will refer to the contact sites *per se* (conceptual definition) as “MERCs”, while “MAMs” will refer to the membranes obtained in the laboratory, where proteins can be detected (operational definition).

Vesicle-associated membrane protein-associated protein B (VAPB) is present at MAMs and reportedly interacts with protein tyrosine phosphatase-interacting protein 51 (PTPIP51), a protein of the outer mitochondrial membrane (OMM). Their interaction tethers ER and mitochondria, thus allowing for efficient Ca^2+^ transfer between both organelles (De Vos et al., 2012). Another tethering protein is GRP75, a MAMs-enriched protein that links the Ca^2+^ channels Inositol 1,4,5-triphosphate receptor (IP3R) at the ER membrane and Voltage-dependent anion channel (VDAC) at the OMM, thereby establishing nanodomains of high Ca^2+^ concentration directly at mitochondrial uptake sites (Szabadkai et al., 2006). Mitofusins (MFNs) are proteins found both in mitochondria and in MAMs, and are proposed to form homodimeric complexes that tether both membranes (Brito and Scorrano, 2008), which can act as a lipid shuttle between both organelles (Hernández-Alvarez et al., 2019). However, that view has been challenged, since MFNs have also been proposed to rather separate ER from mitochondria (Filadi et al., 2015; Puri et al., 2019). Phosphofurin acidic cluster sorting protein 2 (PACS-2) is a ER sorting protein also present in MAMs that, instead of forming bridges, regulates MAMs composition, and its downregulation reportedly decreases ER-mitochondria interaction (Simmen et al., 2005; Myhill et al., 2008).

Although many proteins are reportedly enriched at MAMs, there is no consensus whether there is a unique marker of MERCs. In fact, MERCs composition can vary within the same cell. In HeLa cells, for example, and in response to external cues, Mitofusin-2 (MFN2) levels can increase in either the perinuclear o peripheral regions of the cell, differentally regulating ER-mitochondria proximity between said regions (Bravo-Sagua et al., 2016). This suggests that MERCs are dynamic and heterogeneous structures, broadly distributed within the cell; however, it is currently unknown whether this is true in other cell types. Notwithstanding, skeletal and cardiac myocytes, as we will review in their respective sections, have highly organized and specialized ER and mitochondria distributions. Thus, it is expected that their MERCs are more ordered than in other cell types, where ER and mitochondria do not follow a regular pattern. Due to the nature of the proteins that reside at MAMs, these organelle contacts are relevant for many cell functions, such as maintaining energy balance, calcium (Ca^2+^) regulation, lipid homeostasis, and the regulation of cell death and survival (López-Crisosto et al., 2015).

Among the different forms through which the ER and mitochondria communicate, Ca^2+^ exchange is undoubtedly the most studied process. Ca^2+^ release from the ER and uptake by mitochondria stimulates mitochondrial respiration and ATP production, and thus, is crucial for the regulation of cell bioenergetics (Bravo et al., 2011). This feature is possible because MAMs are dynamic structures that can increase in number and composition, to match the cellular needs (Bravo et al., 2011; Bravo-Sagua et al., 2016; Simmen and Herrera-Cruz, 2018). However, MERCs can also act as a double-edged sword, as they can also trigger mitochondrial collapse and apoptosis. The proteins of the B-cell lymphoma 2 (Bcl-2) family are the primary known regulators of cell death and survival through the ER-mitochondria axis, controlling ER Ca^2+^ leakage (Pinton et al., 2008; Lewis et al., 2014). At the organismal level, deficiency of MAMs-associated proteins can cause different phenotypes, including reduced glucose tolerance and insulin signaling, and also altered lipid metabolism and Ca^2+^ signaling, demonstrating their metabolic relevance in all these mechanisms (Sebastián et al., 2012).

As an organellar interplay, MERCs are crucial for cellular adaptation to their ever-changing environment and inherent changes. For example, stem cells can proliferate according to external cues, yielding two daughter cells: one that remains as a pluri- or multipotent stem cell, and another that acquires specialized characteristics through a differentiation process (Zakrzewski et al., 2019). On the pathological side of proliferating cells stand cancer cells, which undergo uncontrolled proliferation, even under adverse conditions. Of note, along the cell life cycle, MERCs also participate in the generation of reactive oxygen species (ROS), which can either act as signaling molecules or damage cellular structures (Debattisti et al., 2017; Yoboue et al., 2018).

In this review, we aim to show that MERCs also change along cell differentiation; i.e., they also specialize to match the structural and functional needs of cells that perform motor, metabolic, nervous, humoral, and immune responses. Currently, the exact mechanisms that drive MERCs assembly and disassembly during aging and differentiation are yet to be unraveled. Nonetheless, reviewing the known lifecycle of MERCs will shed light on the potential signaling pathways involved. Also, we discuss the best-described aspects of the ER-mitochondrion interplay in physiologic, pathologic and aging scenarios, and we compare how MERCs vary according to cell type and their differentiation state, aiming to understand the changes they undergo along the cellular lifecycle.

## ER-mitochondria communication in proliferating cells

Cell types can be classified according to the degree of cell differentiation. Unlike terminally differentiated cells, stem, and cancer cells share a low grade of differentiation and a high proliferative potential. Although stem cells and cancer cells represent a physiological and pathological dedifferentiation state, respectively, both share features related to their bioenergetic profile, characterized by a high anabolic demand due to their continuous proliferation (Intlekofer and Finley, 2019). Moreover, both cell types largely share a mitochondrial morphology phenotype where fission prevails over fusion, yielding a rather fragmented mitochondrial network (Chen and Chan, 2017). Interestingly, ER-mitochondria contacts are involved in biological processes closely related to the key features observed for stem and cancer cells (Sassano et al., 2017; Zakrzewski et al., 2019).

### Mitochondria-ER contacts and stem cells function

Stem cells specialize in self-renewal by dividing and developing into specialized cells by a tightly regulated asymmetrical cycle of division and differentiation. Stem cells are key components in tissue development and maintenance, and the loss or dysfunction of these cells closely relates to the progression of aging (Vilas et al., 2018). Indeed, stem cells are a highly heterogeneous group of cells, and some of their features are closely related to their degree of differentiation, among other factors. For instance, mitochondrial dynamics regulate the fate and identity of stem cells (Khacho et al., 2016). When mouse embryonic stem cells differentiate, they exhibit a decrease in the levels of the mitochondrial fission protein Dynamin-related protein 1 (DRP1) and mitochondria elongation (Pernaute et al., 2022). Interestingly, using a green fluorescent protein-based contact site sensor for narrow and wide ER and mitochondria juxtaposition, it has been recently shown that mitochondrial fusion promotes ER-mitochondria interaction (Cieri et al., 2018). Therefore, it could be expected that inter-organelles interactions in stem cells depend on the degree of cell differentiation and also, the mitochondrial network status. Noteworthy, the signals that govern stem cells differentiation are closely related to MERCs-associated functions, such as mitochondrial Ca^2+^ influx (Figure 1B). Thus, hematopoietic stem cells (HSCs) exhibit a transition between the exit of quiescence and the entrance to the cell cycle, marked by an increment of the mitochondrial membrane potential (ΔΨm) and mitochondrial Ca^2+^ levels as a result of increased cytoplasmic Ca^2+^ levels (Umemoto et al., 2018). Suppression of the increase in cytoplasmic Ca^2+^ levels with adenosine or nifedipine, as well as Ca^2+^ deprivation in culture, disfavors HSC cell cycle entry (Umemoto et al., 2018). Similarly, in *Drosophila* neural stem cells (NSC) lineages, Ca^2+^ flow from the ER to mitochondria interaction is regulated by the protein Miro-1, which is necessary for NSC function, maintenance, as well as, lineage maturation (Lee et al., 2016).

An element that has been extensively studied in the context of ER-mitochondrial communication is the MFN2 protein, which mediates mitochondrial fusion. MFN2 is present in both the ER and the OMM. MFN2 is required for the differentiation of cardiomyocytes and cortical neurons (Kasahara et al., 2013; Fang et al., 2016). Although cell differentiation is closely related to mitochondrial elongation, MFN2 also regulates other processes. For instance, it has been proposed that MFN2 tethers the ER to mitochondrial surfaces, promoting Ca^2+^ transfer into mitochondria (Brito and Scorrano, 2008; Naon et al., 2016). In this regard, it has been postulated that MFN2-mediated ER-mitochondria tethering promotes Ca^2+^ buffering and subsequent regulation of the nuclear factor of activated T cells (NFAT), a necessary step for the lymphoid differentiation of HSC (Luchsinger et al., 2016). Moreover, the IP3R, a MAMs-resident Ca^2+^ channel, is essential for Calcineurin-dependent differentiation of hematopoietic and cardiac stem cells (Wang et al., 2017). However, we must mention that there is still some controversy regarding the role of MFN2 in ER-reticulum contacts, because in some models it has been described that MFN2 activity antagonizes the ER-mitochondria interaction (Filadi et al., 2015; Puri et al., 2019).

During cell cycle progression, mitochondrial fission/fusion elements are finely tuned: the transitions to G1 and S phases are associate with increased mitochondrial fusion, followed by an increase in the fission components, respectively (Spurlock et al., 2020). In the second half of the cell cycle, mitochondrial fission is instrumental and allows the distribution of the mitochondrial network between daughter cells. Interestingly, a recent article described that mitochondrial fission occurring at the cell midzone depends on ER function and leads to mitochondrial proliferation, whereas mitochondrial division at the cell periphery enables the isolation of damaged material into smaller mitochondria destined for mitophagy (Kleele et al., 2021). Of note, although midzone mitochondrial division requires the ER and actin-mediated mitochondrial pre-constriction, peripheral fission is preceded by lysosomal contact and is regulated by the OMM protein FIS1 (Kleele et al., 2021). Similarly, it has also been recently described the existence of mitochondrial localized pools of the 5′ AMP-activated protein kinase (AMPK) that contribute to energetic stress-induced mitophagy (Drake et al., 2021). However, how these discrete mitochondrial populations and processes are interconnected with MERCs function and how this affects stem cell differentiation and cell cycle progression is currently unknown. This is despite the fact that currently we are fully conscious of the importance of mitochondrial fission, and the possible role of MERCs, in cell proliferation, allowing subsequent cell generations to inherit healthy mitochondria.

### Cancer cells and ER-mitochondrial communication

Tumorigenesis is characterized by different hallmarks, among which the reprogramming of cellular metabolism stands out, since it is necessary to support cell viability and the high proliferation rate produced in a frequently nutrient-poor environment (Pavlova and Thompson, 2016; Senga and Grose, 2021). In this scenario, different elements of the ER-mitochondria interaction remodel to match mitochondrial bioenergetics with the cellular requirements; most notably, the ER-to-mitochondria Ca^2+^ transfer (Kaufman and Malhotra, 2014).

At moderate amounts, ER-to-mitochondria Ca^2+^ transfer promotes tumorigenesis, while excessive transfer leads to cell death, thus acting as a tumor-suppressing mechanism. Thioredoxin-related transmembrane protein 1 (TMX1) is an oxidoreductase enriched in MAMs that decreases the activity of SERCA, the protein that pumps Ca^2+^ into the ER, thus reducing Ca^2+^ levels in the ER. In spite of that, TMX1 is required for ER-mitochondria communication, since its decrease hinders the Ca^2+^ flux between both organelles (Raturi et al., 2016). In HeLa cells, reduction of TMX1 expression promotes tumor growth in athymic nude mice (Raturi et al., 2016). However, TMX1 levels increase in melanoma cell lines (over 70% of the lines tested) compared to primary melanocytes and keratinocytes (Zhang et al., 2019). These data suggest a dual role for this protein, in agreement with the dual role of MERCs in governing cell life and death. Among other examples is TOM70, a MAMs-enriched protein involved in cytosol-to-mitochondria peptide translocation. Reportedly, TOM70 interacts with the Inositol 1,4,5-triphosphate receptor isoform 3 (IP3R3) Ca^2+^ channel at the ER surface. There, it promotes ER-to-mitochondria Ca^2+^ flux, mitochondrial bioenergetics and cell proliferation, thereby highlighting the ER-mitochondria interface as a pro-survival signaling axis (Filadi et al., 2018).

In regard to pharmacological interventions, limitation of basal ER–mitochondria Ca^2+^ flux by inhibition of the IP3R by Xestospongin B (XeB), is mainly toxic for tumor cells (Cárdenas et al., 2016), highlighting the importance of this Ca^2+^ flux for cancer cell survival. MCF7, T47D, and HS578T cell lines and also transformed fibroblast exhibit an exacerbated cell death when treated with XeB (Cárdenas et al., 2016). In contrast, low mitochondrial Ca^2+^ levels lead to a decrease in the tricarboxylic acid (TCA) cycle activity and thus, to the activation of cell survival pathways, such as AMPK-mediated autophagy and the Sirtuin-1 (SIRT1) signaling pathway (Missiroli et al., 2016; Lovy et al., 2020). Interruption of the ER-mitochondria Ca^2+^ flux also affects tumor cells with different levels of oxidative phosphorylation (OXPHOS) dependency. For instance, a common feature of some cancer cells is a reduced level of OXPHOS. Interruption of ER-mitochondria Ca^2+^ transfer in cancer cells with this metabolic profile promotes cell death, which can be attenuated with nucleoside supplementation (Cardenas et al., 2020). On the other hand, cells with a high rate of OXPHOS, such as leukemia cells, are susceptible to the interruption of Ca^2+^ flux in a relatively specific way, since normal lymphocytes remain unaffected (Cruz et al., 2021). In this case, leukemia cell viability is not rescued by nucleosides, accounting for different pathways that lead to cell death (Cruz et al., 2021).

Some cancer cells have altered IP3R3 levels compared to healthy tissues. For example, the expression of this receptor is increased in clear cell renal cell carcinoma, compared to the matched unaffected part of the kidney from the same patient, while the levels of IP3R1 and IP3R2 decrease (Rezuchova et al., 2019). Interestingly, the IP3R1 and IP3R2 isoforms are associated with a pro-apoptotic profile (Lencesova et al., 2013; Bartok et al., 2019; Mo et al., 2021), whereas IP3R3 is associated with proliferative and anti-apoptotic effects (Rezuchova et al., 2019). However, this notion is controversial given the fact that IP3R3-mediated Ca^2+^ fluxes can also promote apoptosis by Ca^2+^ overload (Kuchay et al., 2017; Pulli et al., 2019). In regard to IP3R3, this receptor correlates with the malignancy of different types of cancer, such as cholangiocarcinoma, where its overexpression enhances mitochondrial Ca^2+^ signaling, as well as proliferation and cell migration (Ueasilamongkol et al., 2020). In the same line, demethylation of the *ITPR3* gene and the ensuing increase in IP3R3 protein levels is also frequent in the development of hepatocellular carcinoma (Guerra et al., 2019).

Reportedly, a great diversity of proteins act as regulators of ER-mitochondrial communication. Remarkably, the classical oncoprotein Bcl-2 interacts with IP3R through its BH4 domain, and acts as an inhibitor of ER Ca^2+^ release (Guerra et al., 2019). In turn, in hepatocellular carcinoma cells, MFN2 overexpression promotes apoptosis by increasing ER–mitochondria Ca^2+^ flux (Wang et al., 2015b). In these examples, Ca^2+^ overload favors apoptosis and, to a certain extent, opposes tumorigenesis. All this evidence reveals a complex relationship between MERCs and cancer progression, which apparently, depends on the cell type.

Besides regulating Ca^2+^transport, MAMs also contain a significant number of enzymes related to cholesterol and phospholipid traffick and metabolism (Poston et al., 2013; Vance, 2014). In cancer, it is common to observe changes in choline and ethanolamine phospholipids, and drugs that target enzymes involved in their synthesis have been proposed as probable pharmacological cancer treatments (Cheng et al., 2016). In mice, hepatic MFN2 deficiency reduces phosphatidylserine transfer from the ER to the mitochondria, causing ER stress and liver damage that can lead to cancer (Hernández-Alvarez et al., 2019). In the case of cholesterol, Caveolin-1 (CAV1), a cell surface protein that is also enriched in MAMs, drives cholesterol traffic from the ER to the plasma membrane through the secretory pathway (Simón et al., 2020). Reportedly, CAV1 hinders ER-mitochondria communication, rendering cells prone to cell death upon ER stress, and thus, acts as a tumor suppressor (Bravo-Sagua et al., 2018). This observation agrees with the notion that MERCs are required for cell adaptation to adverse scenarios.

MERCs also influence the immune system’s recognition of malignant glioma stem-like cells (GSC). For instance, in a glioma cell line (GL261) neurosphere derivative, an artificial tether promotes surface glycan expression, suggesting that MERCs regulate surface expression of sialylated glycans that later modulate the cytotoxic T cells responses (Bassoy et al., 2017). Further, GSC lines show highly dynamic mitochondrial networks in comparison to glioma differentiated cells (GDC) lines. Thus, mitochondria dynamic interventions in GSC lines modulate susceptibility to immune response (Bassoy et al., 2017). Interestingly, it has been reported that alterations in mitochondrial dynamics modify the number of MERCs (Cieri et al., 2018). Through the use of reporters like the split-GFP-based contact site sensor (SPLICS), it has been shown that ER-mitochondria contacts of different sizes can behave differently when faced with the same stimulus (Cieri et al., 2018).

### Senescent cells and Mitochondria-ER contacts

Cell senescence is a mechanism whereby a dividing cell enters a stable cell cycle arrest and becomes unresponsive to the mitogenic and apoptotic signals triggered by stressing stimulus or their proliferative limit (Hernandez-Segura et al., 2018). Moreover, cellular senescence is a mechanism that counteracts the growth of cancer cells. Senescent cells in culture may develop a “flattened” appearance, a larger size with an abnormal expansion of their nucleus and an alteration in lysosomal biogenesis, which is observed by staining as an accumulation of granules in the cytoplasm. These granules are a response to an increase in senescence-associated beta-galactosidase (SA-β-gal) activity (Spazzafumo et al., 2017). Senescent cells are also characterized by cell cycle restriction through increased expression of molecules such as p16, p53, and p21 activity (Hernandez-Segura et al., 2018). Additionally, senescent cells increase the secretion of inflammatory and chemoattractant factors, which facilitate their recognition and removal by the immune system. This signaling mechanism is known as the senescent-associated secretory phenotype, or SASP (Wiley et al., 2021). Therefore, cell senescence acts as a mechanism whereby a dividing cell enters a stable cell cycle arrest upon a stressing stimulus, while remaining metabolically active through the SASP, and unresponsive to mitogenic and apoptotic signals (Hernandez-Segura et al., 2018). Physiologically, aging and exposure to different stress stimuli progressively limit the ability of the immune system to handle senescent cells, which accumulate and promote the development of metabolic disorders (Ferrucci and Fabbri, 2018).

Attention has turned to the role of MERC in the development and maintenance of cellular senescence, since their increase leads to exacerbated Ca^2+^ transfer and, ultimately, Ca^2+^ overload in mitochondria. This phenomenon increases the production of ROS (Figure 1B) and might be beneficial in overcoming the resistance of senescent cells to apoptotic pathways (Madreiter-Sokolowski et al., 2018; Cavinato et al., 2021). Therefore, the modulation of the communication between these two organelles looks like an interesting alternative in the study of cellular senescence and normal cell turnover and tissue homeostasis. For instance, in human mammary epithelial cells, IP3R2, and MCU, both related to ER-mitochondria Ca^2+^ modulation, directly impact senesce induction. Thus, IP3R2 knockdown boost oncogene-induced senescence escape, while IP3R2 activation promotes premature senescence (Wiel et al., 2014). Similarly, in primary cultures of rat hippocampal neurons, it has been shown that increased days in culture (to resemble an *in vitro* model of aging) promote an increase in MCU expression and ER-mitochondria Ca^2+^ transfer (Calvo-Rodríguez et al., 2016). Moreover, the authors of this work propose that Ca^2+^ remodeling may contribute to an aging-related cognitive decline and neuron cell death (Calvo-Rodríguez et al., 2016).

In liver cells, both *in vitro* and *in vivo*, inhibition of IP3R2 increases the distance between mitochondria and the ER, i.e., disassembly of MERCs. As a result, the SA-β-GAL and p16 markers of cell senescence decrease, thus preventing age-related hepatic fibrosis and steatosis. In contrast, a shorter distance between the ER and mitochondrial contacts promotes cell senescence through increased mitochondrial Ca^2+^ and ROS production, cell cycle restriction through induction of p53, and a NFκB-dependent SASP phenotype (Ziegler et al., 2021b). Further, in human endometrium-derived stem cells (hMESC) sublethal H_2_O_2_- treatment resulted in a rapid Ca^2+^ release from intracellular stores mediated by the activation of an IP3R-dependent pathway that later results in the development of a senescent phenotype. This phenotype is accompanied by persistently elevated intracellular Ca^2+^ levels. Interestingly, Ca^2+^ chelation with BAPTA-(AM) was sufficient to prevent the expansion of hMESCs senescence (Borodkina et al., 2016).

## ER-mitochondria communication in differentiated cells

MERCs also play a critical role in fully differentiated cells. On the one hand, metabolism is a key aspect that governs the function of contractile cells, such as cardiomyocytes, skeletal muscle and vascular smooth muscle cells (Rohlenova et al., 2018; Gandoy-Fieiras et al., 2020; Shi et al., 2020; Gastel and Carmeliet, 2021). On the other hand, MERCs are very dynamic structures that are sensitive to metabolic changes. For example, in HeLa cells we demonstrated that early ER stress increases mitochondria-ER interactions (Bravo et al., 2011). Also, using split-GFP MERCs reporters it has been shown that energy stress favors the formation of MERCs in an AMPK-dependent manner (Hu et al., 2020). These antecedents highlight the importance of MERCs in the physiology and pathophysiology of differentiated cells. Another proposed idea is that MERCs also play a fundamental role in the immune response. Since MERCs regulate Ca^2+^ homeostasis, they may regulate lymphocyte activation, which is a process that requires Ca^2+^ signaling (Martinvalet, 2018). The following section discusses the function of MERCs in terminally differentiated cells and its potential role in their aging.

### ER-mitochondria communication in endothelial and vascular smooth muscle cells

The vasculature mainly comprises endothelial cells and vascular smooth muscle cells (VSMC). On the one hand, endothelial cells are key regulators of vascular tone, angiogenesis, blood fluidity, and inflammation (Jia et al., 2019). On the other hand, VSMC and the extracellular matrix generated by these cells mediate the contractile tone of arteries, therefore regulating blood pressure, as well as modulating blood flow and blood vessel repair (Chi et al., 2018).

**FIGURE 2:**
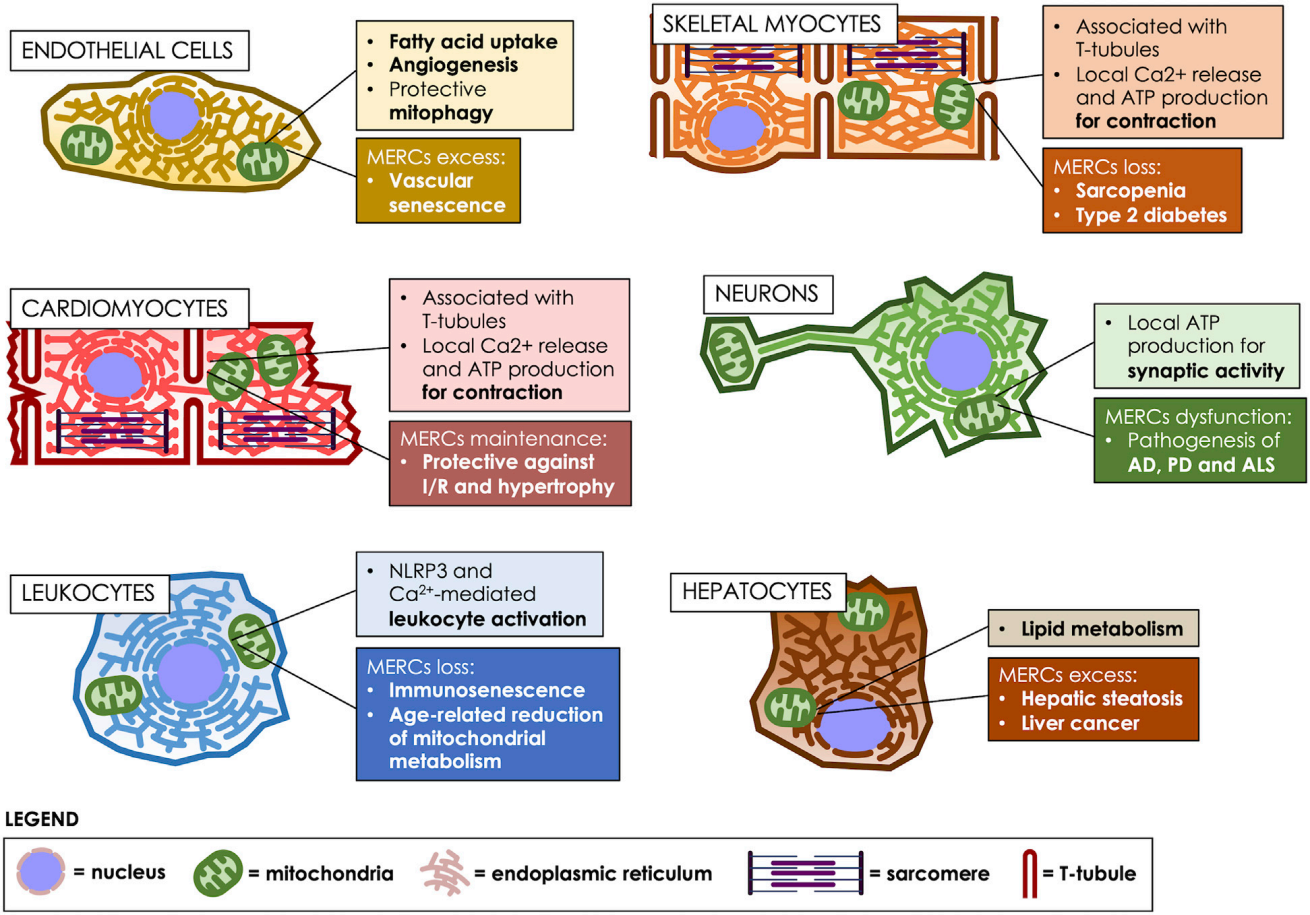
MERCs fulfill various roles, according to the cell type. MERCs differentiate both in structure and function, according to the cell’s differentiation state, participating in crucial processes such as motor, nervous, metabolic and immunological responses. Age-related changes in MERCs are heavily implicated in the development of multiple pathologies. T-tubules: transverse tubules.

As reported, MERCs are relevant in vascular physiology and pathophysiology (Figure 2). For instance, MERCs participate in endothelial uptake and transport of fatty acids (FA). FA uptake requires locally-produced ATP by mitochondria, and is mediated by the fatty acid transport protein 4 (FATP4) (Ibrahim et al., 2020). Moreover, this transporter is present in the ER, and FA uptake is driven by its ATP-dependent acyl-CoA synthetase activity (Ibrahim et al., 2020). These results suggest that MERCs can mediate the transport of circulating FA uptake through the endothelium to reach other tissues. In a pathophysiological context, a recent study reported that the α2 agonist dexmedetomidine reduced vascular permeability in a caecal ligation and puncture model of sepsis in rats, as well as in primary vascular endothelial cells stimulated with lipopolysaccharide (She et al., 2021). Interestingly, this study showed that dexmedetomidine would exert its protective effect in sepsis through a mechanism involving increased expression of ZO-1, Occludin and VE-cadherin, inhibition of mitochondrial fission, and reduced MERCs by modulating the polymerization of actin stress fibers dependent on the α2 receptor (She et al., 2021).

FUN14 domain-containing protein 1 (FUNDC1) is an OMM protein involved in the generation of MERCs that participates in angiogenesis, as revealed by *in vitro* and *in vitro* experiments (Wang et al., 2021). Impaired formation of MERCs by silencing FUNDC1 decreases spheroid-sprouting tube formation (a well-established assay to assess angiogenesis) and VEGFR2 levels (Wang et al., 2021). These findings suggest that MERCs induced by FUNDC1 may be a novel therapeutic target for the treatment of diseases that involve pathological neovascularization.

The role of MERCs in VSMC mitophagy (Moulis et al., 2019) was evaluated through studies that showed that PACS-2 accumulates in MAMs in response to oxidized LDL, and its silencing associates with defective mitophagosome formation, thereby promoting VSMC apoptosis (Moulis et al., 2019). Therefore, these findings suggest that the PACS-2 MERCs protein is an attractive target to limit the risk of atherosclerotic plaque rupture.

In the context of aging, senescent endothelial cells may contribute to impaired vascular tone, altered endothelium permeability and angiogenesis; thus, endothelial cell senescence is considered a risk factor in cardiovascular diseases associated with age (Jia et al., 2019). With regard to VSMC, cellular senescence is associated with reduced sensibility to contracting or relaxing stimuli and a VSMC phenotype switch from contractile to synthetic (Jia et al., 2019).

*In vitro*, aged endothelial cells show an increase of MERCs, thus promoting mitochondrial Ca^2+^ uptake and metabolism, which may be associated with increased susceptibility of senescent endothelial cells to death by Ca^2+^ overload (Madreiter-Sokolowski et al., 2018). Furthermore, treatment of rat aortic endothelial cells with angiotensin II elicits premature senescence, increased mitochondrial fragmentation, and monocyte adhesion (Miyao et al., 2020). Interestingly, treatment with the mitochondrial division inhibitor-1 (mdivi-1) reversed these effects, suggesting a potential therapeutic role of mitochondrial fission repression, and the subsequently decrease in senescence and inflammation generated by angiotensin II (Miyao et al., 2020). Nonetheless, further research is required to thoroughly establish the link between mitochondrial fission and the senescence-associated inflammatory phenotype and eventually, explore its translational value in the context of vascular diseases. However, the potential targeting of MERCs and senescence in the context of disease has already been explored. Thus, Kim et al. (2018) reported that Protein disulfide isomerase A1 (PDIA1) -involved in protein folding in the ER- can act as a thiol reductase for DRP1. Knockdown of PDIA1 in endothelial cells elicited senescence, and promoted sulfenylation of DRP1 at Cys644, thereby inducing mitochondrial fission and ROS production, without triggering ER stress. Interestingly, impaired wound healing was observed in PDIA1^+/−^ or diabetic mice, but restoration of the endothelial PDIA1-DRP1 pathway reverted this effect (Kim et al., 2018). Considering that DRP1 locates at MERCs during fission, targeting this axis could reduce MERCs, which consequently would reduce mitochondrial fission. This highlights the translational value of targeting endothelial MERCs to limit senescence and thereby, mitigate diabetic vasculature complications.

Overall, MERCs reportedly play an important role in physiological and pathophysiological processes in vascular cells. Furthermore, the aforementioned studies indicate that a link between MERCs and senescence in these cells is being explored, but research in this field is still in its early stages.

### Structure and function of Mitochondria-ER contacts in skeletal myocytes

Skeletal muscle makes up 40% of the body’s muscle mass and is an energy-consuming organ essential for physiological performance. The basic unit of muscle cells are the sarcomeres, which are composed of an array of contractile proteins that assemble as linear fibers, interleaved by traverse tubules, which are invaginations of the plasma membrane, called sarcolemma in these specialized cells (Helle et al., 2013; Rebelo et al., 2020). Sarcomeres are surrounded by mitochondria, which provide the ATP required for sarcomere contraction, and by ER membranes, known as sarco/endoplasmic reticulum (SR) in these cells, which are in charge of releasing the Ca^2+^ that triggers contraction (Blaauw et al., 2017; Kim et al., 2018). In these cells, MERCs play a crucial role in orchestrating Ca^2+^ storage, energy production and protein folding (Rebelo et al., 2020) (Figure 2). Therefore, depletion of MERCs is associated with pathologies such as muscular dystrophy and age-related diseases such as insulin resistance (Zhang et al., 2021b). Thus, a proteomic analysis of rabbit skeletal muscle determined 459 proteins enriched in MAMs, which consisted in mitochondrial, SR and transverse-tubules proteins; therefore, this study revealed that in these cells, MERCs are specialized compartments that directly interact with the sarcolemma (Liu et al., 2015).

Altered Ca^2+^ homeostasis and transfer between the ER and mitochondria are implicated in the muscle dysfunction observed in aging (Zhang et al., 2021b). This dysfunction is mainly produced by a reduced mitochondrial Ca^2+^ uptake, which explains the decline in muscle performance associated with advanced age (Pietrangelo et al., 2015). During aging, a disrupted SR-mitochondria Ca^2+^ transport could play a role in muscle dysfunction through mitochondrial Ca^2+^ homeostasis impairment. SR-mitochondria interaction during aging is reduced in skeletal muscle, which is associated with the downregulation of SR-mitochondria Ca^2+^ transport proteins IP3R1, VDAC1, and GRP75; the last one, which tethers IP3R with VDAC1 (Xu et al., 2018; Gill et al., 2019). Interestingly, exercise improves this condition (Zhang et al., 2021b). Moreover, sarcopenia is a loss of skeletal muscle mobility and physical capacity in aging that is also characterized by a SR-mitochondria loss of communication. This disease can, in turn, lead to other pathological conditions, such as a decreased metabolic rate, increased insulin resistance and loss of bone mass (Cederholm and Morley, 2015; Del Campo et al., 2018).

As shown in mouse muscle fibers, proteins governing mitochondrial dynamics change in a non-linear fashion during aging, which correlates with changes in ER-mitochondrial communication and mitochondrial Ca^2+^ handling in the aged muscle (Del Campo et al., 2018). Furthermore, altered MERCs relate to impaired skeletal muscle metabolic function (Cárdenas-Pérez and Camacho, 2016). In addition, a study in the gastrocnemius muscle of obese mice with type 2 diabetes showed a deficiency of SR-mitochondria contacts similar to what is observed in human patients with obesity and diabetes (Tubbs et al., 2018). For these reasons, recent studies have been directed to evaluate the role of MERCs in type 2 diabetes mellitus, in the search for new possible pharmacological targets (Tubbs et al., 2018).

SEPN1-related myopathy is an autosomal recessive congenital muscle disease due to inherited defects in Selenoprotein N1 (SEPN1), an ubiquitous ER/SR protein that protects against oxidative stress and is involved in Ca^2+^ homeostasis (Filipe et al., 2021). In an *in vivo* model, SEPN1 knockout mice showed marked alterations in mitochondrial physiology and energy metabolism, suggesting that SEPN1 controls mitochondrial bioenergetics. Moreover, SEPN1 is enriched at MAMs, and is required for Ca^2+^ transients between the SR and mitochondria and the integrity of MERCs. Consistently, loss of SEPN1 in human patients associates with alterations in body composition, which correlate with the severity of the disease-associated muscle weakness, impaired SR-mitochondria contacts, and lower ATP levels (Filipe et al., 2021).

### Mitochondria-ER contacts and cardiomyocytes

Similarly to skeletal muscle cells, the structure of cardiomyocytes consists of an array of sarcomeres containing the contractile myofibrils interrupted by transverse tubules. Mitochondria occupy ∼40% of the total cardiomyocyte volume, which is two-fold higher than in skeletal muscle fibers. Notably, in cardiac muscle, ∼90% of the Ca^2+^ release units of the SR are close to mitochondria, mainly located at MAMs (Sharma et al., 2000; Eisner et al., 2013). In cardiac and skeletal myocytes, the mitochondrial Ca^2+^ uniporter (MCU) is an inner mitochondrial membrane protein that allows Ca^2+^ entry to the mitochondrial matrix after Ca^2+^ diffusion across the OMM *via* VDACs; the isoforms VDAC1-3 (Modesti et al., 2021). Reportedly, Ca^2+^ conductance of the uniporter is much higher in skeletal-muscle-derived than in cardiac-muscle-derived mitoplasts (Fieni et al., 2012). Similarly, the mRNA levels of the MCU and mitochondrial calcium uptake 1 (MICU1), an essential regulator of the MCU, were also higher in skeletal-muscle-derived mitoplasts than in those derived from cardiac muscle (Perocchi et al., 2010; De Stefani et al., 2011).

CVDs are the leading cause of morbidity and mortality worldwide, including cardiomyopathy, heart failure (HF), hypertension, and atherosclerosis (Li et al., 2019; Tang et al., 2020). In this aspect, aging is one of the causes of increased CVDs. Aged hearts exhibit DNA damage, ER stress, mitochondrial dysfunction, contractile dysfunction, hypertrophic growth, and SASP (Tang et al., 2020). Consequently, cardiomyocyte senescence is involved in aging, heart dysfunction and HF. Notably, the dysfunction and senescence of stressed and aged cardiomyocytes also involve non-myocyte-derived factors through the release of proinflammatory signals (Tang et al., 2020). In parallel, the deficiency of proteins, such as carnitine-palmitoyl transferase-1 (CPT1), peroxisome proliferator-activated receptor α (PPARα), and PGC-1α, among others, aggravates cardiomyocyte senescence since they decrease with age, thus leading to cardiomyocyte growth and hypertrophy (Tang et al., 2020). Moreover, in a model of cardiomyocyte hypertrophy induced by norepinephrine, our group found increased mitochondrial fission (Pennanen et al., 2014), together with a decreased functional and physical communication between the ER and mitochondria, which directly impacted insulin signaling, thus causing insulin desensitization (Gutiérrez et al., 2014).

The ER and mitochondria are also essential organelles in the regulation of protein homeostasis to maintain cardiomyocyte function and survival (Figure 2). As already mentioned, the loss of interaction between the ER and mitochondria in cardiomyocytes increases with aging and heart disease, thereby decreasing mitochondrial function and altering cellular processes such as oxidative stress, autophagy and metabolic imbalance, consequently affecting the overall heart function (Murley and Nunnari, 2016; Gude et al., 2018). For instance, alterations in the ER-mitochondria interaction result in the progression of acute myocardial ischemia/reperfusion (I/R) (Cheng et al., 2021) and diabetic heart disease (Dia et al., 2020; Zhang et al., 2021a), due to ER and mitochondria stress, which ultimately decrease mitochondrial function (Gude et al., 2018). Mitochondrial Ca^2+^ uptake is decreased in aged cardiomyocytes, and this has been associated with a decreased production of NADPH and increased generation of mitochondrial ROS. Thus, this defective ER-mitochondrial communication in aged hearts leads to alterations in Ca^2+^ transfer from the ER to the mitochondria (Fernandez-Sanz et al., 2014; Li et al., 2019). Moreover, and also in the aging heart, the altered SR-mitochondria interaction indirectly affects autophagy, apoptosis, and mitochondrial dysfunction, ultimately leading to cardiac dysfunction. However, the mechanisms involved in this specific process have not been fully elucidated yet (Li et al., 2019), which can be one of the reasons why no optimal drug therapy for HF and atrial fibrillation is currently available.

Remarkably, melatonin exerts cardioprotective effects in several cardiovascular disorders (Zhou et al., 2018). Furthermore, melatonin is protective against cardiomyocyte I/R injury by stabilizing MERCs, modulating gene expression, and mediating mitochondrial protection by preventing the positive regulation of the IP3R in I/R (Li et al., 2021).

### Mitochondria-ER contacts and their relationship with neuronal function

In the brain, the aging process associates with a decrease in the adaptive capacity and loss of homeostasis, which involves the accumulation of senescent cells, including dysfunctional neurons and senescent neural stem cells, microglia, astrocytes, and oligodendrocytes (Sikora et al., 2021). The accumulation of senescent cells leads to degenerative changes that favor a SASP-mediated progressive cognitive decline that disrupts the synapse, and the structural and functional interaction between neurons and glial cells (Han et al., 2020). These alterations have been related to the development of neurodegenerative diseases, such as Alzheimer’s (AD), Parkinson’s (PD), and Amyotrophic Lateral Sclerosis (ALS). Some of the proposed mechanisms to explain neurodegeneration include altered protein dynamics, inflammation, changes in Ca^2+^ homeostasis, oxidative stress, ROS production, and dysfunction of mitochondrial bioenergetics.

An optimal metabolic capacity is essential for efficient cell function; however, impaired energy metabolism is inherent to aging. The deterioration of OXPHOS, which occurs in the early stages of cell senescence, increases the production of ROS, and promotes nuclear and mitochondrial DNA damage (Srivastava, 2017). A lower ΔΨm and ATP production impair cell function, facilitating neurodegeneration. Furthermore, the modulation of mitochondrial dynamics is decisive in responding to mitochondrial DNA damage, alterations of proteostasis and accumulation of ROS (Srivastava, 2017). Since MERCs are essential regulators of mitochondrial dynamics, the study of their participation in the modulation of aging and neurodegeneration is highly relevant (Figure 2).

In astrocytes, MERCs are mainly located in cellular regions termed perivascular endfeet, where astrocyte-vasculature communication takes place (Göbel et al., 2020). Following brain injury, these zones are responsible for astrocyte-driven neo-vascularization as an adaptive response; and during this regenerative process, perivascular endfeet are theorized to have high energy demands to cope with intercellular communication. Astrocyte-specific MERCs disruption through MFN2 deletion severely impairs vascular remodeling, indicating that ER-mitochondria contacts are essential for astrocytic regenerative signaling (Göbel et al., 2020). In neurons, MERCs are also present in zones of high energy demand, the pre- and postsynaptic regions, where they modulate local changes in Ca^2+^ concentration, and mitochondrial dynamics (Shirokova et al., 2020). Lowering DRP1 protein levels reportedly decreases MERCs, causes mitochondrial elongation and reduces mitochondrial presence at the terminal synaptic boutons of long motor neurons, thereby leading to locomotor deficits in *D. melanogaster*. Recovery of DRP1 expression restores the number of MERCs, mitochondrial fission and axonal distribution, as well as neuron function (Fowler et al., 2020). Taking into account that DRP1-mediated mitochondrial constriction occurs at MERCs, this evidence suggests that MERCs support neurotransmission.

As recently proposed, alterations of MERCs would be an important element in the development of neurodegenerative disorders. Different components of the unfolded protein response (UPR) machinery, including the PERK, IRE1α and ER chaperones associated with neurodegenerative disorders, are found in MAMs (Leal and Martins, 2021). PERK is necessary for the proper docking of ER and mitochondria and the ROS-induced apoptotic response. Cells deficient in PERK exhibit increased basal and peak respiration and increased ROS production, impaired mtDNA biogenesis, and impaired apoptosis (Cullinan and Diehl, 2004; Zheng et al., 2012; Munoz et al., 2013; Rainbolt et al., 2014), together with fewer MERCs (Verfaillie et al., 2012; Leal and Martins, 2021).

AD is characterized by the accumulation of amyloid-beta (Aβ) peptides, which form extracellular plaques. Amyloid precursor protein (APP) is processed by the MAMs-enriched γ-secretase complex proteins, presenilin 1 (PS1) and presenilin 2 (PS2). AD-linked mutant forms of these proteins lead to abnormal cleavages of APP, producing the aggregation-prone Aβ forms Aβ40 and Aβ42, the latter being the major component of amyloid plaques (Area-Gomez et al., 2009). Changes in MAMs proteome occur in the early stages of Aβ accumulation, suggesting that MERCs are involved in the pathogenesis of AD (Völgyi et al., 2018). Animal models of AD have reported alterations of MERC, with consequences in the deterioration of lipid and energy metabolism (Liu and Zhu, 2017).

In the case of ALS, alterations in the expression of MAMs proteins are responsible for its pathogenesis. These alterations would compromise the integrity of MERCs by increasing Ca^2+^ flow to the mitochondria (Chen et al., 2021). For instance, and consistent with a role of MERCs in ALS, mutations in the gene-encoding VAPB have been found in cases of familial ALS (Nishimura et al., 2004; Fasana et al., 2010). VAPB controls multiple functions in central nervous system cells, including protein transport, lipid metabolism, autophagy, and mitochondrial Ca^2+^ entry (Vos et al., 2012; Murphy and Levine, 2016; Gomez-Suaga et al., 2017). Thus, the consequence of an ALS VAPB mutant is a reduction of mitochondrial OXPHOS, which normally depends on Ca^2+^ import from the ER for its dehydrogenases and the ATPase function (Fan and Simmen, 2019). A second example is the protein VCP, which is responsible for 1–2% of familial ALS cases (Johnson et al., 2010). VCP acts as an ubiquitin-specific chaperone, controlling proteasomal protein degradation at the ER (Wójcik et al., 2006), mitochondria (Xu et al., 2011), and lysosomes (Papadopoulos et al., 2017). On MERCs, VCP interacts with the E3 ubiquitin ligase Gp78/AMFR, which in addition to its role in ER-associated degradation, also determines MERC function by controlling the amounts of MFN2 (Wang et al., 2015a; Li et al., 2015) and the degradation of HMG-CoA reductase, one of the enzymes of the mevalonate/cholesterol pathway (Song et al., 2005). Thus, VCP controls cholesterol levels at the ER, which are required for MERC cholesterol raft formation (Sano et al., 2009). In the presence of a mutant VCP protein, MERCs are dysfunctional due to altered cholesterol levels. Moreover, the mutation of VCP disrupts the control of OXPHOS normally maintained by Ca^2+^ and ROS signaling on MERCs, as observed in ALS patient fibroblasts (Bartolome et al., 2013).

Disruption of MERCs has also been associated with Parkinson’s disease (PD). Under normal conditions, the α-synuclein, Parkin and PTEN-induced kinase (PINK) proteins are concentrated in MAMs, while mutations in these proteins have been associated with disturbances of mitochondrial-ER communication (Ziegler et al., 2021a). Likewise, the absence of Parkin can explain a relative resistance to mitophagy in neurons, thus favoring the accumulation of dysfunctional mitochondria and neuronal damage (Kook et al., 2020; Lopez-Crisosto et al., 2021). The mitochondrial GTPase Miro-1 is a protein located in MAMs that is involved in mitochondrial axonal trafficking. Miro-1 participates in the regulation of Ca^2+^ homeostasis, in mitochondrial quality control mediated by PINK1/Parkin, and in the regulation of mitochondrial transport, which is necessary for neuronal survival. Alterations in Miro-1 functioning have been reported to result in perinuclear clustering of mitochondria, altered mitophagy, and mitochondrial trafficking, thereby affecting MERCs quality (Tang, 2015). Cellular studies have demonstrated that a mutation in Miro-1 increases the number of dysfunctional MERCs, showing that its dysregulation interferes with mitophagy in different PD cell models (Berenguer-Escuder et al., 2020). Interestingly, alterations of MERCs are related to neurodegenerative diseases with different characteristics. Therefore, more studies are required to understand the mechanisms involved in MERCS alterations, in order to establish the bases for early therapeutic interventions in the initial stages of neurodegenerative disorders.

### Leukocytes and ER-mitochondria interaction

The progressive deterioration of the immune response associated with aging or immunosenescence is a low-grade chronic inflammatory state (Oh et al., 2019). The alterations of the immune response that occur with increasing age are associated with a decrease in the response to vaccines, greater susceptibility to infections and the development of neurodegenerative diseases (Oh et al., 2019). Immunosenescence is characterized by a decrease in the number of naïve cells and an increase in memory cells. A decrease in the number of macrophages, neutrophils with diminished phagocytic capacity and alterations in the function of monocytes have also been reported in aged people (Aiello et al., 2019; Oh et al., 2019). In relation to adaptive immunity, there is a lower response of B cells to new antigens, a greater accumulation of senescent memory T cells and a decrease in naïve T cells. These changes generate a decrease in the immune response and an increase of inflammatory signals (Aiello et al., 2019).

The inflammasomes are protein sensor complexes responsible for initiating the inflammatory response as a protective mechanism against damage (Christgen and Kanneganti, 2020). The inflammasome comprises a caspase activating complex that triggers an inflammatory response with the release of interleukins and the induction of cell death; in addition, it is an important modulator of inflammatory changes associated with age (Christgen and Kanneganti, 2020). Activation of the inflammasome requires recognition receptors, which include Toll-like receptors (TLRs), RIG-I-like receptors, lectin C-like receptors (CLR), and NOD-like receptors (NLR). NLRs, such as NLRP1, NLRP3, NLRC4 are expressed in most tissues. NLRP3 associates with the ER membranes and, after activation, moves to the MAMs, initiating the inflammasome activity (Escoll et al., 2017). As reported, NLPR3 activation responds to Ca^2+^ fluxes, increased mitochondrial ROS, and increased mitochondrial fission (Yang and Jafri, 2020).

MERCS are key in the regulation of immunity, as immune functions are highly dependent on lipid transfer, autophagosome formation, mitochondrial fission, and Ca^2+^ flux. In this sense, intracellular Ca^2+^ flux is essential for lymphocyte activation, so dysregulation of Ca^2+^ homeostasis may affect lymphocyte activation (Martinvalet, 2018). MERCs are involved in the modulation of cellular Ca^2+^ homeostasis, which suggest that MERCs most likely play a critical role in modulating lymphocyte activation. Parallel, leukocyte migration depends on changes in mitochondrial dynamics and Ca^2+^ levels, both also regulated by MERCs. In fact, a redistribution of mitochondria and MERCs occurs in leukocytes during both of these processes, providing a platform for the recruitment of cytoskeletal proteins that allow immune cell chemotaxis (Martinvalet, 2018).

Ca^2+^ signaling is necessary for lymphocyte activation and neutrophil chemotaxis; thus, increases in mitochondrial Ca^2+^ stimulate ATP production, suggesting that MERCs are necessary during the activation of the immune system (Yang and Jafri, 2020). Once faced with the inflammatory response, the immune cells need to migrate to carry out innate and adaptive responses, which in turn requires that they reorganize their fission-dependent mitochondrial network. Because T cells undergo mitochondrial fission more frequently, they present smaller mitochondria that can be seen scattered throughout the cytoplasm (Breda et al., 2019; Xie et al., 2020).

Some studies have shown that the transfer of Ca^2+^ from the ER through the IP3R channels to the mitochondria and its subsequent mitochondrial accumulation leads to cellular senescence in normal human cells (Ziegler et al., 2021b). For instance, and although the role of IP3R2 in Ca^2+^ transfer and cellular senescence is unknown, this channel has been reported to promote some features associated with age and aging in mice (Ziegler et al., 2021b). IP3R2 KO mouse models have shown a lower number of CD4^+^ memory T cells and CD8^+^ effector memory T cells when compared to the control groups, supporting a role for IP3R2 in promoting immunosenescence. Likewise, it has been reported that the loss of IP3R2 decreases the level of senescence *in vitro* and *in vivo*, suggesting a regulatory role in senescence and aging (Ziegler et al., 2021b). In parallel, the reduction of MERCs in mice by ablation of the IP3R2 reduces the number of senescent cells and the expression of SASP (Ziegler et al., 2021b; Ghosh-Choudhary et al., 2021). However, more studies are required to elucidate whether these effects are due to reduced mitochondrial Ca^2+^ overload, decreased ER-mitochondrial contacts, or a combination of both mechanisms. Interestingly, lymphocyte activation also requires IP3-mediated intracellular Ca^2+^ flux, so regulation of Ca^2+^ homeostasis through MERCs is a common modulator of the immune response (Martinvalet, 2018).

Changes in mitochondrial bioenergetics can be reflected in the respiratory profiles of circulating cells. Peripheral blood mononuclear cells (PBMC) have been proposed as biomarkers of the whole-body mitochondrial profile because they are readily available cells, and their oxidative metabolism correlates well with certain pathologies (Karabatsiakis et al., 2014; Herpich et al., 2020). In this regard, studies have shown that the respiratory capacity of PBMC decreases with age (Bektas et al., 2019; Callender et al., 2020). Moreover, deterioration of the mitochondrial function of monocytes from elderly patients occurs during maximum respiration, which suggests that lower respiratory capacity occurs in the face of high energy demands, such as those produced in inflammatory processes (Bektas et al., 2019; Callender et al., 2020).

PBMC include cell types responsible for essential functions of the inflammatory response. Aging associates with deterioration of the immune response, which could be due to mitochondrial and MERCs dysfunction. The mechanisms underlying these alterations are not entirely clear, but they are related to alterations of mitochondrial dynamics and the dysfunction of OXPHOS (Pence and Yarbro, 2018). Therefore, the deregulation of MERCs can affect cell homeostasis, favoring cell damage and, in turn, disease development. Since mitochondria and the ER are responsible for the regulation of Ca^2+^ through the MERC, this results in ROS generation, whose imbalance drives oxidative damage capable of triggering cellular senescence. However, the role of MERCs and their mechanism in cell senescence has not been completely elucidated in PBMCs yet.

### Mitochondria-ER contacts in hepatocytes

MERCs also allow the flow of macronutrients and micronutrients between organelles. Lipids and Ca^2+^ are important for oxidative metabolism in the mitochondria, allowing energy production, while in a pathological context, they have been associated with greater permeability of the inner membrane and increased oxidative stress (Marchi et al., 2018). For instance, the flux of lipids between the ER and the mitochondria-mediated by MAMs proteins such as MFN2, is essential for the synthesis and oxidation of mitochondrial lipids. In C57BL6/J mice fed a high-fat diet, Mfn2 expression decreased, while hepatic steatosis increased, indicating that lipid transfer to mitochondria is fundamental to prevent their accumulation (Hernández-Alvarez et al., 2019). Indeed, the hepatic absence of Mfn2 in mice fed with a regular diet increased the accumulation of hepatic triglycerides and decreased the hepatic phospholipid content (Hernández-Alvarez et al., 2019). More recently, it has been also showed that a Mfn2 deficiency in mice liver, reduces phosphatidylserine transfer and phospholipids synthesis, leading to ER stress, the development of a NASH-like phenotype and subsequently, liver cancer (Anastasia et al., 2021; Ziegler et al., 2021b). Thus, it seems that MERCs coordinate Ca^2+^ transfer between the ER and mitochondria through the physical interaction between VDAC1 and IP3R. In this regard, it has been described that the alteration in Ca^2+^ levels would be responsible for the cell damage observed in NASH, by increasing the expression of IP3R (Ma et al., 2021). However, the exact role of ER-mitochondrial communication in hepatic metabolism remains unresolved.

Interestingly, studies in mouse hepatocytes have further evidenced contacts between mitochondria and the rough endoplasmic reticulum and their role in VLDL and hepatic fatty acid secretion, lipid droplets and lipid content. Thereby the existence of the wrappER-mitochondria contact, which corresponds to the contact between the rough ER and mitochondria, has been described recently in mouse hepatocytes (Ma et al., 2021). The wrappER-mitochondrial contact would function as a site for VLDL biogenesis. Ablation of the wrappER-mitochondrial contact decreases VLDL secretion, resulting in systemic changes in lipids in mice (Ma et al., 2021).

Additionally, the downregulation of MERCs prevents the development of steatosis and liver cirrhosis, while hepatocytes from obese animals revealed an increased number of MERCs (Arruda et al., 2014; Ziegler et al., 2021a). As reported, artificial induction of MERCs only affected hepatocytes when animals developed obesity, leading to the conclusion that an increased number of MERCs and overweight cause the mitochondrial dysfunction that affects liver metabolism (Arruda et al., 2014). However, these results encountered some dissidence, because another study indicated that increased MERCs improves liver function (Tubbs et al., 2014), as it has been described in other tissues (Gutiérrez et al., 2014; Dia et al., 2020; Cheng et al., 2021). This discrepancy could be explained by several reasons: 1. The difference in the nutritional status of the animals; 2. The environmental factors that can alter the microbiota of animals and the status of MERCS; 3. The different analyses of the integrity of the MERCs and, 4. The areas of the liver that were analyzed. In summary, while Tubbs et al., did not consider mitochondrial density, to conclude that increased MERCs improve glucose metabolism (Tubbs et al., 2014); Arruda et al. (2014) did not address the stress caused by the adenoviral tool used to increased MERCs in hepatocytes Finally, both authors differ in the experimental conditions used, which can finally influence the reported conclusions, as discussed by (Rieusset, 2018).

Regarding Ca^2+^ signaling, the contribution of MERCs has been poorly studied in hepatocytes. IP3R1 and IP3R2 are the dominant intracellular Ca^2+^ channels, while IP3R3 is less expressed (Guerra et al., 2019). However, there is increased IP3R3 expression in hepatocellular carcinoma related to less survival of patients, while its suppression enhances apoptosis of the cancer cells (Guerra et al., 2019).

## Conclusion and future perspectives

MERCs structure and function vary along a cell’s life cycle, starting from their undifferentiated state. In proliferating cells, they are instrumental for proper mitochondrial distribution between daughter cells, acting as scaffolds for mitochondrial fission. Later on, they participate in the differentiation process, acting as regulators of intracellular Ca^2+^ signals. Their plasticity is also important for adaptation to changes in the extracellular milieu. However, it is a double-edged sword, since exacerbation of MERCs function can either lead to cell senescence or death. Thus, in cancer cells the relationship between MERCs and cell viability is complex: they must either be enhanced, to promote cell survival, or decreased, to prevent mitochondrial Ca^2+^ overload and subsequent cell demise. In differentiated cells, MERCs are also specialized to cope with the cellular emerging functions. These functions vary greatly, ranging from 1) FA uptake and angiogenesis in the vasculature, 2) local Ca^2+^ and ATP providers at the sites of contraction in skeletal and cardiac myocytes, forming a triumvirate with the sarcolemma, 3) coordinators of mitochondrial location at synaptic termini, as well as potential culprits of neurodegeneration, 4) activators of immune responses in leukocytes, to 5) metabolic hubs for lipid metabolism in the liver.

In summary, MERCs have their own life cycle and differentiation pathways, leading to an interesting variety of structural and functional phenotypes. Moreover, they not only participate in normal, physiological processes, but also in the pathological, dysfunctional states of differentiated (and dedifferentiated) cells. These results are not surprising, since MERCs are not an organelle *per se*, but rather a subcompartment that harmonizes the activities of two major organelle networks: the ER and mitochondria. Such is their importance, that MERCs are reportedly altered in all the pathologies in which they have been studied. Therefore, further and thorough investigations of their functions are required to fully understand the progression of different diseases. Moreover, MERCs pose a pivotal therapeutic target for age-related maladies, a topic that has been addressed only recently. Because of their complexity, it is probable that cell-specific MERCs-targeted therapeutic approaches must also be explored for them to be efficient and safe.

## Glossary

AD: Alzheimer’s disease
ALS: Amyotrophic Lateral Sclerosis
Aβ: amyloid-beta
AMPK: AMP-ctivated protein kinase
APP: Amyloid precursor protein
ATP: adenosine triphosphate
Bcl-2: B-cell lymphoma 2 (Bcl-2) protein
BH: Bcl-2 homology
Ca2+: Calcium
CAV1: Caveolin-1
CLR: Lectin C-like receptors
CPT1: Carnitine-palmitoyl transferase-1
CVDs: Cardiovascular diseases
DNA: Deoxyribonucleic acid
DRP1: Dynamin-related protein 1
ER: Endoplasmic reticulum
FA: fatty acids
FIS1: Mitochondrial fission 1 protein
FUNDC1: FUN14 domain-containing protein 1
Gp78/AMFR: Tumor autocrine motility factor receptor
Grp75: glucose-regulated protein 75
GSC: Glioma stem-like cells
HF: Heart failure
HMG-CoA reductase: 3-Hydroxy-3-Methylglutaryl-CoA Reductase
hMESCs: human endometrium‐derived stem cells
HSCs: Hematopoietic stem cells
I/R: ischemia/reperfusion
IP3R: Inositol 1,4,5-triphosphate receptor
IRE1α: Inositol-requiring enzyme-1α
ITPR3: Inositol 1,4,5-trisphosphate receptor type 3 human gene
LDL: Low-density lipoprotein
MAMs: Mitochondria-associated membranes
MCU: Mitochondrial calcium uniporter; mdivi-1: mitochondrial division inhibitor 1
MERCs: Mitochondria-ER contact sites
MFNs: Mitofusins
MICU1: Mitochondrial calcium uptake 1
Miro-1: Mitochondrial Rho GTPases; NADPH: Nicotinamide adenine dinucleotide phosphate
NSC: Neural stem cells
NFAT: Nuclear factor of activated T cells
NFκB: Nuclear factor kappa B
NLR: NOD-like receptors
OMM: Outer mitochondrial membrane
OXPHOS: Oxidative phosphorylation
PACS-2: Phosphofurin acidic cluster sorting protein 2
PBMCs: Peripheral blood mononuclear cells
PD: Parkinson’s disease
PDIA1: protein disulfide isomerase A1
PERK: Protein kinase R (PKR)-like endoplasmic reticulum kinase
PGC-1α: Peroxisome proliferator-activated receptor gamma coactivator 1 alpha
PINK: PTEN-induced kinase 1
PPARα: Peroxisome proliferator-activated receptor α
PTPIP51: Protein tyrosine phosphatase-interacting protein 51
RIG-I-like receptors: Retinoic acid-inducible gene I-like receptor
ROS: Reactive oxygen species
RyR2: Ryanodine receptor calcium channel 2
SASP: Senescent-associated secretory phenotype
SA-β-gal: Senescence-associated beta-galactosidase
SEPN1: Selenoprotein N
SERCA: Sarco/endoplasmic reticulum Ca2+-ATPase
SIRT1: Silent mating type information regulation 2 homolog 1
SR: sarcoplasmic reticulum
TCA: Tricarboxylic acid
TLR: Toll-like receptors
TMX1: Thioredoxin-related transmembrane protein 1
TOM70: Translocase of outer mitochondrial membrane 70
UPR: Unfolded protein response
VAPB: Vesicle-associated membrane protein-associated protein B
VCP: Valosin-containing protein
VDAC1: voltage-dependent anion channel 1
VEGFR2: Vascular endothelial growth factor receptor 2
VSMC: Vascular smooth muscle cells
XeB: Xestospongin B
ZO-1: Zonula occludens 1
ΔΨm: Mitochondria membrane potential

## References

[B1] Aiello A. Farzaneh F. Candore G. Caruso C. Davinelli S. Gambino C. M. (2019). Immunosenescence and its hallmarks: How to oppose aging strategically? A review of potential options for therapeutic intervention. Front. Immunol. 10, 2247. 10.3389/fimmu.2019.02247 31608061 PMC6773825

[B2] Anastasia I. Ilacqua N. Raimondi A. Lemieux P. Ghandehari-Alavijeh R. Faure G. (2021). Mitochondria-rough-ER contacts in the liver regulate systemic lipid homeostasis. Cell Rep. 34, 108873. 10.1016/j.celrep.2021.108873 33730569

[B3] Aoyama-Ishiwatari S. Hirabayashi Y. (2021). Endoplasmic reticulum–mitochondria contact sites—emerging intracellular signaling hubs. Front. Cell Dev. Biol. 9, 653828. 10.3389/fcell.2021.653828 34095118 PMC8172986

[B4] Area-Gomez E. Groof A. J. C. D. Boldogh I. Bird T. D. Gibson G. E. Koehler C. M. (2009). Presenilins are enriched in endoplasmic reticulum membranes associated with mitochondria. Am. J. Pathol. 175, 1810–1816. 10.2353/ajpath.2009.090219 19834068 PMC2774047

[B5] Arruda A. P. Pers B. M. Parlakgül G. Güney E. Inouye K. Hotamisligil G. S. (2014). Chronic enrichment of hepatic endoplasmic reticulum-mitochondria contact leads to mitochondrial dysfunction in obesity. Nat. Med. 20, 1427–1435. 10.1038/nm.3735 25419710 PMC4412031

[B6] Bartok A. Weaver D. Golenár T. Nichtova Z. Katona M. Bánsághi S. (2019). IP3 receptor isoforms differently regulate ER-mitochondrial contacts and local calcium transfer. Nat. Commun. 10, 3726. 10.1038/s41467-019-11646-3 31427578 PMC6700175

[B7] Bartolome F. Wu H.-C. Burchell V. S. Preza E. Wray S. Mahoney C. J. (2013). Pathogenic VCP mutations induce mitochondrial uncoupling and reduced ATP levels. Neuron 78, 57–64. 10.1016/j.neuron.2013.02.028 23498975 PMC3843114

[B8] Bassoy E. Y. Kasahara A. Chiusolo V. Jacquemin G. Boydell E. Zamorano S. (2017). ER–mitochondria contacts control surface glycan expression and sensitivity to killer lymphocytes in glioma stem‐like cells. Embo J. 36, 1493–1512. 10.15252/embj.201695429 28283580 PMC5452011

[B9] Bektas A. Schurman S. H. Gonzalez-Freire M. Dunn C. A. Singh A. K. Macian F. (2019). Age-associated changes in human CD4+ T cells point to mitochondrial dysfunction consequent to impaired autophagy. Aging 11, 9234–9263. 10.18632/aging.102438 31707363 PMC6874450

[B10] Berenguer-Escuder C. Grossmann D. Antony P. Arena G. Wasner K. Massart F. (2020). Impaired mitochondrial–endoplasmic reticulum interaction and mitophagy in Miro1-mutant neurons in Parkinson’s disease. Hum. Mol. Genet. 29, 1353–1364. 10.1093/hmg/ddaa066 32280985 PMC7254851

[B11] Blaauw B. Schiaffino S. Reggiani C. (2017). Mechanisms modulating skeletal muscle phenotype. Compr. Physiol. 3, 1645–1687. 10.1002/cphy.c130009 24265241

[B12] Borodkina A. V. Shatrova A. N. Deryabin P. I. Griukova A. A. Abushik P. A. Antonov S. M. (2016). Calcium alterations signal either to senescence or to autophagy induction in stem cells upon oxidative stress. Aging 8, 3400–3418. 10.18632/aging.101130 27941214 PMC5270676

[B13] Bravo R. Vicencio J. M. Parra V. Troncoso R. Munoz J. P. Bui M. (2011). Increased ER-mitochondrial coupling promotes mitochondrial respiration and bioenergetics during early phases of ER stress. J. Cell Sci. 124, 2143–2152. 10.1242/jcs.080762 21628424 PMC3113668

[B14] Bravo-Sagua R. López-Crisosto C. Parra V. Rodriguez-Peña M. Rothermel B. A. Quest A. F. G. (2016). mTORC1 inhibitor rapamycin and ER stressor tunicamycin induce differential patterns of ER-mitochondria coupling. Sci. Rep. 6, 36394. 10.1038/srep36394 27808250 PMC5093439

[B15] Bravo-Sagua R. Parra V. Sandoval C. O. Navarro-Marquez M. Rodriguez A. E. Diaz-Valdivia N. (2018). Caveolin-1 impairs PKA-DRP1-mediated remodelling of ER-mitochondria communication during the early phase of ER stress. Cell Death Differ. 50, 1195–1212. 10.1038/s41418-018-0197-1 PMC6748148 30209302

[B16] Breda C. N. de S. Davanzo G. G. Basso P. J. Câmara N. O. S. Moraes-Vieira P. M. M. (2019). Mitochondria as central hub of the immune system. Redox Biol. 26, 101255. 10.1016/j.redox.2019.101255 31247505 PMC6598836

[B17] Brito O. M. D. Scorrano L. (2008). Mitofusin 2 tethers endoplasmic reticulum to mitochondria. Nature 456, 605–610. 10.1038/nature07534 19052620

[B18] Callender L. A. Carroll E. C. Bober E. A. Akbar A. N. Solito E. Henson S. M. (2020). Mitochondrial mass governs the extent of human T cell senescence. Aging Cell 19, e13067. 10.1111/acel.13067 31788930 PMC6996952

[B19] Calvo-Rodríguez M. García-Durillo M. Villalobos C. Núñez L. (2016). *In vitro* aging promotes endoplasmic reticulum (ER)-mitochondria Ca2+ cross talk and loss of store-operated Ca2+ entry (SOCE) in rat hippocampal neurons. Biochim. Biophys. Acta 1863, 2637–2649. 10.1016/j.bbamcr.2016.08.001 27503411

[B20] Cardenas C. Lovy A. Silva-Pavez E. Urra F. Mizzoni C. Ahumada-Castro U. (2020). Cancer cells with defective oxidative phosphorylation require endoplasmic reticulum–to–mitochondria Ca2+ transfer for survival. Sci. Signal. 13, eaay1212. 10.1126/scisignal.aay1212 32665411 PMC9387586

[B21] Cárdenas C. Müller M. McNeal A. Lovy A. Jaña F. Bustos G. (2016). Selective vulnerability of cancer cells by inhibition of Ca(2+) transfer from endoplasmic reticulum to mitochondria. Cell Rep. 14, 2313–2324. 10.1016/j.celrep.2016.02.030 26947070 PMC4794382

[B22] Cárdenas-Pérez R. E. Camacho A. (2016). Roles of calcium and Mitochondria-Associated Membranes in the development of obesity and diabetes. Med. Univ. 18, 23–33. 10.1016/j.rmu.2015.10.004

[B23] Cavinato M. Madreiter‐Sokolowski C. T. Büttner S. Schosserer M. Zwerschke W. Wedel S. (2021). Targeting cellular senescence based on interorganelle communication, multilevel proteostasis, and metabolic control. Febs J. 288, 3834–3854. 10.1111/febs.15631 33200494 PMC7611050

[B24] Cederholm T. Morley J. E. (2015). Sarcopenia: The new definitions. Curr. Opin. Clin. Nutr. Metab. Care 18, 1–4. 10.1097/mco.0000000000000119 25207791

[B25] Chen H. Chan D. C. (2017). Mitochondrial dynamics in regulating the unique phenotypes of cancer and stem cells. Cell Metab. 26, 39–48. 10.1016/j.cmet.2017.05.016 28648983 PMC5539982

[B26] Chen J. Bassot A. Giuliani F. Simmen T. (2021). Amyotrophic lateral sclerosis (ALS): Stressed by dysfunctional mitochondria-endoplasmic reticulum contacts (MERCs). Cells 10, 1789. 10.3390/cells10071789 34359958 PMC8304209

[B27] Cheng D. Zheng J. Hu F. Lv W. Lu C. (2021). Abnormal mitochondria-endoplasmic reticulum communication promotes myocardial infarction. Front. Physiol. 12, 717187. 10.3389/fphys.2021.717187 34413791 PMC8369510

[B28] Cheng M. Bhujwalla Z. M. Glunde K. (2016). Targeting phospholipid metabolism in cancer. Front. Oncol. 6, 266. 10.3389/fonc.2016.00266 28083512 PMC5187387

[B29] Chi C. Li D.-J. Jiang Y.-J. Tong J. Fu H. Wu Y.-H. (2018). Vascular smooth muscle cell senescence and age-related diseases: State of the art. Biochim. Biophys. Acta. Mol. Basis Dis. 1865, 1810–1821. 10.1016/j.bbadis.2018.08.015 31109451

[B30] Christgen S. Kanneganti T.-D. (2020). Inflammasomes and the fine line between defense and disease. Curr. Opin. Immunol. 62, 39–44. 10.1016/j.coi.2019.11.007 31837596 PMC7067632

[B31] Cieri D. Vicario M. Giacomello M. Vallese F. Filadi R. Wagner T. (2018). Splics: A split green fluorescent protein-based contact site sensor for narrow and wide heterotypic organelle juxtaposition. Cell Death Differ. 25, 1131–1145. 10.1038/s41418-017-0033-z 29229997 PMC5988678

[B32] Cruz P. Ahumada-Castro U. Bustos G. Molgó J. Sauma D. Lovy A. (2021). Inhibition of InsP3R with Xestospongin B reduces mitochondrial respiration and induces selective cell death in T cell acute lymphoblastic leukemia cells. Int. J. Mol. Sci. 22, 651. 10.3390/ijms22020651 PMC7827595 33440859

[B33] Csordás G. Weaver D. Hajnóczky G. (2018). Endoplasmic reticulum–mitochondrial contactology: Structure and signaling functions. Trends Cell Biol. 28, 523–540. 10.1016/j.tcb.2018.02.009 29588129 PMC6005738

[B34] Cullinan S. B. Diehl J. A. (2004). PERK-Dependent activation of Nrf2 contributes to redox homeostasis and cell survival following endoplasmic reticulum stress. J. Biol. Chem. 279, 20108–20117. 10.1074/jbc.m314219200 14978030

[B35] De Stefani D. Raffaello A. Teardo E. Szabò I. Rizzuto R. (2011). A forty-kilodalton protein of the inner membrane is the mitochondrial calcium uniporter. Nature 476, 336–340. 10.1038/nature10230 21685888 PMC4141877

[B36] De Vos K. J. Mórotz G. M. Stoica R. Tudor E. L. Lau K.-F. Ackerley S. (2012). VAPB interacts with the mitochondrial protein PTPIP51 to regulate calcium homeostasis. Hum. Mol. Genet. 21, 1299–1311. 10.1093/hmg/ddr559 22131369 PMC3284118

[B37] Debattisti V. Gerencser A. A. Saotome M. Das S. Hajnóczky G. (2017). ROS control mitochondrial motility through p38 and the motor adaptor miro/trak. Cell Rep. 21, 1667–1680. 10.1016/j.celrep.2017.10.060 29117569 PMC5710826

[B38] Del Campo A. Contreras-Hernández I. Castro-Sepúlveda M. Campos C. A. Figueroa R. Tevy M. F. (2018). Muscle function decline and mitochondria changes in middle age precede sarcopenia in mice. Aging 10, 34–55. 10.18632/aging.101358 29302020 PMC5811241

[B39] Dia M. Gomez L. Thibault H. Tessier N. Leon C. Chouabe C. (2020). Reduced reticulum–mitochondria Ca2+ transfer is an early and reversible trigger of mitochondrial dysfunctions in diabetic cardiomyopathy. Basic Res. Cardiol. 115, 74. 10.1007/s00395-020-00835-7 33258101 PMC7704523

[B40] Drake J. C. Wilson R. J. Laker R. C. Guan Y. Spaulding H. R. Nichenko A. S. (2021). Mitochondria-localized AMPK responds to local energetics and contributes to exercise and energetic stress-induced mitophagy. Proc. Natl. Acad. Sci. U. S. A. 118, e2025932118. 10.1073/pnas.2025932118 34493662 PMC8449344

[B41] Eisner V. Csordás G. Hajnóczky G. (2013). Interactions between sarco-endoplasmic reticulum and mitochondria in cardiac and skeletal muscle - pivotal roles in Ca²⁺ and reactive oxygen species signaling. J. Cell Sci. 126, 2965–2978. 10.1242/jcs.093609 23843617 PMC3711195

[B42] Escoll P. Rolando M. Buchrieser C. (2017). MAMs are attractive targets for bacterial repurposing of the host cell: MAM-functions might be key for undermining an infected cell. BioEssays 39, 1600171. 10.1002/bies.201600171 28026026

[B43] Fan Y. Simmen T. (2019). Mechanistic connections between endoplasmic reticulum (ER) redox control and mitochondrial metabolism. Cells 8, 1071. 10.3390/cells8091071 PMC6769559 31547228

[B44] Fang D. Yan S. Yu Q. Chen D. Yan S. S. (2016). Mfn2 is required for mitochondrial development and synapse formation in human induced pluripotent stem cells/hiPSC derived cortical neurons. Sci. Rep. 6, 31462. 10.1038/srep31462 27535796 PMC4989148

[B45] Fasana E. Fossati M. Ruggiano A. Brambillasca S. Hoogenraad C. C. Navone F. (2010). A VAPB mutant linked to amyotrophic lateral sclerosis generates a novel form of organized smooth endoplasmic reticulum. Faseb J. 24, 1419–1430. 10.1096/fj.09-147850 20008544

[B46] Fernandez-Sanz C. Ruiz-Meana M. Miro-Casas E. Nuñez E. Castellano J. Loureiro M. (2014). Defective sarcoplasmic reticulum–mitochondria calcium exchange in aged mouse myocardium. Cell Death Dis. 5, e1573. 10.1038/cddis.2014.526 25522267 PMC4454162

[B47] Ferrucci L. Fabbri E. (2018). Inflammageing: Chronic inflammation in ageing, cardiovascular disease, and frailty. Nat. Rev. Cardiol. 15, 505–522. 10.1038/s41569-018-0064-2 30065258 PMC6146930

[B48] Fieni F. Lee S. B. Jan Y. N. Kirichok Y. (2012). Activity of the mitochondrial calcium uniporter varies greatly between tissues. Nat. Commun. 3, 1317. 10.1038/ncomms2325 23271651 PMC3818247

[B49] Filadi R. Greotti E. Turacchio G. Luini A. Pozzan T. Pizzo P. (2015). Mitofusin 2 ablation increases endoplasmic reticulum–mitochondria coupling. Proc. Natl. Acad. Sci. U. S. A. 112, E2174–E2181. 10.1073/pnas.1504880112 25870285 PMC4418914

[B50] Filadi R. Leal N. S. Schreiner B. Rossi A. Dentoni G. Pinho C. M. (2018). TOM70 sustains cell bioenergetics by promoting IP3R3-mediated ER to mitochondria Ca2+ transfer. Curr. Biol. 28, 369–382. e6. 10.1016/j.cub.2017.12.047 29395920

[B51] Filipe A. Chernorudskiy A. Arbogast S. Varone E. Villar-Quiles R.-N. Pozzer D. (2021). Defective endoplasmic reticulum-mitochondria contacts and bioenergetics in SEPN1-related myopathy. Cell Death Differ. 28, 123–138. 10.1038/s41418-020-0587-z 32661288 PMC7853070

[B52] Fowler P. C. Byrne D. J. Blackstone C. O'Sullivan N. C. (2020). Loss of the mitochondrial fission GTPase Drp1 contributes to neurodegeneration in a Drosophila model of hereditary spastic paraplegia. Brain Sci. 10, 646. 10.3390/brainsci10090646 PMC7564485 32957716

[B53] Gandoy-Fieiras N. Gonzalez-Juanatey J. R. Eiras S. (2020). Myocardium metabolism in physiological and pathophysiological states: Implications of epicardial adipose tissue and potential therapeutic targets. Int. J. Mol. Sci. 21, 2641. 10.3390/ijms21072641 PMC7177518 32290181

[B54] Gastel N. V. Carmeliet G. (2021). Metabolic regulation of skeletal cell fate and function in physiology and disease. Nat. Metab. 3, 11–20. 10.1038/s42255-020-00321-3 33398192

[B55] Ghosh‐Choudhary S. K. Liu J. Finkel T. (2021). The role of mitochondria in cellular senescence. Faseb J. 35, e21991. 10.1096/fj.202101462r 34758157 PMC8720272

[B56] Gill J. F. Delezie J. Santos G. McGuirk S. Schnyder S. Frank S. (2019). Peroxisome proliferator‐activated receptor γ coactivator 1α regulates mitochondrial calcium homeostasis, sarcoplasmic reticulum stress, and cell death to mitigate skeletal muscle aging. Aging Cell 18, e12993. 10.1111/acel.12993 31290266 PMC6718523

[B57] Gӧbel J. Engelhardt E. Pelzer P. Sakthivelu V. Jahn H. M. Jevtic M. (2020). Mitochondria-endoplasmic reticulum contacts in reactive astrocytes promote vascular remodeling. Cell Metab. 31, 791–808. e8. 10.1016/j.cmet.2020.03.005 32220306 PMC7139200

[B58] Gomez-Suaga P. Paillusson S. Stoica R. Noble W. Hanger D. P. Miller C. C. J. (2017). The ER-mitochondria tethering complex VAPB-PTPIP51 regulates autophagy. Curr. Biol. 27, 371–385. 10.1016/j.cub.2016.12.038 28132811 PMC5300905

[B59] Gude N. A. Broughton K. M. Firouzi F. Sussman M. A. (2018). Cardiac ageing: Extrinsic and intrinsic factors in cellular renewal and senescence. Nat. Rev. Cardiol. 15, 523–542. 10.1038/s41569-018-0061-5 30054574

[B60] Guerra M. T. Florentino R. M. Franca A. Filho A. C. L. Santos M. L. D. Fonseca R. C. (2019). Expression of the type 3 InsP3 receptor is a final common event in the development of hepatocellular carcinoma. Gut 68, 1676–1687. 10.1136/gutjnl-2018-317811 31315892 PMC7087395

[B61] Gutiérrez T. Parra V. Troncoso R. Pennanen C. Contreras-Ferrat A. Vasquez-Trincado C. (2014). Alteration in mitochondrial Ca2+ uptake disrupts insulin signaling in hypertrophic cardiomyocytes. Cell Commun. Signal. 12, 68. 10.1186/s12964-014-0068-4 25376904 PMC4234850

[B62] Han X. Zhang T. Liu H. Mi Y. Gou X. (2020). Astrocyte senescence and alzheimer’s disease: A review. Front. Aging Neurosci. 12, 148. 10.3389/fnagi.2020.00148 32581763 PMC7297132

[B63] Helle S. C. J. Kanfer G. Kolar K. Lang A. Michel A. H. Kornmann B. (2013). Organization and function of membrane contact sites. Biochim. Biophys. Acta 1833, 2526–2541. 10.1016/j.bbamcr.2013.01.028 23380708

[B64] Hernández-Alvarez M. I. Sebastián D. Vives S. Ivanova S. Bartoccioni P. Kakimoto P. (2019). Deficient endoplasmic reticulum-mitochondrial phosphatidylserine transfer causes liver disease. Cell 177, 881–895. e17. 10.1016/j.cell.2019.04.010 31051106

[B65] Hernandez-Segura A. Nehme J. Demaria M. (2018). Hallmarks of cellular senescence. Trends Cell Biol. 28, 436–453. 10.1016/j.tcb.2018.02.001 29477613

[B66] Herpich C. Franz K. Klaus S. Müller-Werdan U. Ost M. Norman K. (2020). Age-related fatigue is associated with reduced mitochondrial function in peripheral blood mononuclear cells. Exp. Gerontol. 144, 111177. 10.1016/j.exger.2020.111177 33279665

[B67] Hu Y. Chen H. Zhang L. Lin X. Li X. Zhuang H. (2020). The AMPK-MFN2 axis regulates MAM dynamics and autophagy induced by energy stresses. Autophagy 17, 1142–1156. 10.1080/15548627.2020.1749490 32249716 PMC8143230

[B68] Ibrahim A. Yucel N. Kim B. Arany Z. (2020). Local mitochondrial ATP production regulates endothelial fatty acid uptake and transport. Cell Metab. 32, 309–319. e7. 10.1016/j.cmet.2020.05.018 32521232 PMC7415739

[B69] Intlekofer A. M. Finley L. W. S. (2019). Metabolic signatures of cancer cells and stem cells. Nat. Metab. 1, 177–188. 10.1038/s42255-019-0032-0 31245788 PMC6594714

[B70] Jia G. Aroor A. R. Jia C. Sowers J. R. (2019). Endothelial cell senescence in aging-related vascular dysfunction. Biochim. Biophys. Acta. Mol. Basis Dis. 1865, 1802–1809. 10.1016/j.bbadis.2018.08.008 31109450

[B71] Johnson J. O. Mandrioli J. Benatar M. Abramzon Y. Deerlin V. M. V. Trojanowski J. Q. (2010). Exome sequencing reveals VCP mutations as a cause of familial ALS. Neuron 68, 857–864. 10.1016/j.neuron.2010.11.036 21145000 PMC3032425

[B72] Karabatsiakis A. Böck C. Salinas-Manrique J. Kolassa S. Calzia E. Dietrich D. E. (2014). Mitochondrial respiration in peripheral blood mononuclear cells correlates with depressive subsymptoms and severity of major depression. Transl. Psychiatry 4, e397. 10.1038/tp.2014.44 PMC4080325 26126180

[B73] Kasahara A. Cipolat S. Chen Y. Dorn G. W. Scorrano L. (2013). Mitochondrial fusion directs cardiomyocyte differentiation via calcineurin and notch signaling. Science 342, 734–737. 10.1126/science.1241359 24091702

[B74] Kaufman R. J. Malhotra J. D. (2014). Calcium trafficking integrates endoplasmic reticulum function with mitochondrial bioenergetics. Biochim. Biophys. Acta 1843, 2233–2239. 10.1016/j.bbamcr.2014.03.022 24690484 PMC4285153

[B75] Khacho M. Clark A. Svoboda D. S. Azzi J. MacLaurin J. G. Meghaizel C. (2016). Mitochondrial dynamics impacts stem cell identity and fate decisions by regulating a nuclear transcriptional Program. Cell Stem Cell 19, 232–247. 10.1016/j.stem.2016.04.015 27237737

[B76] Kim Y.-M. Youn S.-W. Sudhahar V. Das A. Chandhri R. Grajal H. C. (2018). Redox regulation of mitochondrial fission protein drp1 by protein disulfide isomerase limits endothelial senescence. Cell Rep. 23, 3565–3578. 10.1016/j.celrep.2018.05.054 29924999 PMC6324937

[B77] Kleele T. Rey T. Winter J. Zaganelli S. Mahecic D. Lambert H. P. (2021). Distinct fission signatures predict mitochondrial degradation or biogenesis. Nature 593, 435–439. 10.1038/s41586-021-03510-6 33953403

[B78] Kook S. Zhan X. Thibeault K. Ahmed M. R. Gurevich V. V. Gurevich E. V. (2020). Mdm2 enhances ligase activity of parkin and facilitates mitophagy. Sci. Rep. 10, 5028. 10.1038/s41598-020-61796-4 32193420 PMC7081349

[B79] Kuchay S. Giorgi C. Simoneschi D. Pagan J. Missiroli S. Saraf A. (2017). PTEN counteracts FBXL2 to promote IP3R3- and Ca2+-mediated apoptosis limiting tumour growth. Nature 546, 554–558. 10.1038/nature22965 28614300 PMC5627969

[B80] Leal N. S. Martins L. M. (2021). Mind the gap: Mitochondria and the endoplasmic reticulum in neurodegenerative diseases. Biomedicines 9, 227. 10.3390/biomedicines9020227 33672391 PMC7926795

[B81] Lee S. Lee K.-S. Huh S. Liu S. Lee D.-Y. Hong S. H. (2016). Polo kinase phosphorylates Miro to control ER-mitochondria contact sites and mitochondrial Ca2+ homeostasis in neural stem cell development. Dev. Cell 37, 174–189. 10.1016/j.devcel.2016.03.023 27093086 PMC4839004

[B82] Lencesova L. Hudecova S. Csaderova L. Markova J. Soltysova A. Pastorek M. (2013). Sulphide signalling potentiates apoptosis through the up‐regulation of IP3 receptor types 1 and 2. Acta Physiol. 208, 350–361. 10.1111/apha.12105 23582047

[B83] Lewis A. Hayashi T. Su T.-P. Betenbaugh M. J. (2014). Bcl-2 family in inter-organelle modulation of calcium signaling; roles in bioenergetics and cell survival. J. Bioenerg. Biomembr. 46, 1–15. 10.1007/s10863-013-9527-7 24078116 PMC4529064

[B84] Li J. Zhang D. Brundel B. J. J. M. Wiersma M. (2019). Imbalance of ER and mitochondria interactions: Prelude to cardiac ageing and disease? Cells 8, 1617. 10.3390/cells8121617 PMC6952992 31842269

[B85] Li L. Gao G. Shankar J. Joshi B. Foster L. J. Nabi I. R. (2015). p38 MAP kinase–dependent phosphorylation of the Gp78 E3 ubiquitin ligase controls ER–mitochondria association and mitochondria motility. Mol. Biol. Cell 26, 3828–3840. 10.1091/mbc.e15-02-0120 26337390 PMC4626067

[B86] Li W. Liu B. Wang L. Liu J. Yang X. Zheng J. (2021). Melatonin attenuates cardiac ischemia-reperfusion injury through modulation of IP3R-mediated mitochondria-ER contact. Oxid. Med. Cell. Longev. 2021, 1370862. 10.1155/2021/1370862 34422206 PMC8371645

[B87] Liao Y. Dong Y. Cheng J. (2020). The molecular determinants of mitochondrial membrane contact with ER, lysosomes and peroxisomes in neuronal physiology and pathology. Front. Cell. Neurosci. 14, 194. 10.3389/fncel.2020.00194 32848610 PMC7427582

[B88] Liu Y. Zhu X. (2017). Endoplasmic reticulum-mitochondria tethering in neurodegenerative diseases. Transl. Neurodegener. 6, 21. 10.1186/s40035-017-0092-6 28852477 PMC5567882

[B89] Liu Z. Du X. Deng J. Gu M. Hu H. Gui M. (2015). The interactions between mitochondria and sarcoplasmic reticulum and the proteome characterization of mitochondrion‐associated membrane from rabbit skeletal muscle. Proteomics 15, 2701–2704. 10.1002/pmic.201400493 25781153

[B90] López-Crisosto C. Bravo-Sagua R. Rodriguez-Peña M. Mera C. Castro P. F. Quest A. F. G. (2015). ER-to-mitochondria miscommunication and metabolic diseases. Biochim. Biophys. Acta 1852, 2096–2105. 10.1016/j.bbadis.2015.07.011 26171812

[B91] Lopez-Crisosto C. Díaz-Vegas A. Castro P. F. Rothermel B. A. Bravo-Sagua R. Lavandero S. (2021). Endoplasmic reticulum−mitochondria coupling increases during doxycycline-induced mitochondrial stress in HeLa cells. Cell Death Dis. 12, 657. 10.1038/s41419-021-03945-9 34183648 PMC8238934

[B92] López-Crisosto C. Pennanen C. Vásquez-Trincado C. Morales P. E. Bravo-Sagua R. Quest A. F. G. (2017). Sarcoplasmic reticulum-mitochondria communication in cardiovascular pathophysiology. Nat. Rev. Cardiol. 14, 342–360. 10.1038/nrcardio.2017.23 28275246

[B93] Lovy A. Ahumada-Castro U. Bustos G. Farias P. Gonzalez-Billault C. Molgó J. (2020). Concerted action of AMPK and sirtuin-1 induces mitochondrial fragmentation upon inhibition of Ca2+ transfer to mitochondria. Front. Cell Dev. Biol. 8, 378. 10.3389/fcell.2020.00378 32523953 PMC7261923

[B94] Luchsinger L. L. Almeida M. J. de Corrigan D. J. Mumau M. Snoeck H.-W. (2016). Mitofusin 2 maintains haematopoietic stem cells with extensive lymphoid potential. Nature 529, 528–531. 10.1038/nature16500 26789249 PMC5106870

[B95] Ma X. Qian H. Chen A. Ni H.-M. Ding W.-X. (2021). Perspectives on mitochondria–ER and mitochondria–lipid droplet contact in hepatocytes and hepatic lipid metabolism. Cells 10, 2273. 10.3390/cells10092273 34571924 PMC8472694

[B96] Madreiter-Sokolowski C. T. Waldeck-Weiermair M. Bourguignon M.-P. Villeneuve N. Gottschalk B. Klec C. (2018). Enhanced inter-compartmental Ca2+ flux modulates mitochondrial metabolism and apoptotic threshold during aging. Redox Biol. 20, 458–466. 10.1016/j.redox.2018.11.003 30458321 PMC6243020

[B97] Marchi S. Patergnani S. Missiroli S. Morciano G. Rimessi A. Wieckowski M. R. (2018). Mitochondrial and endoplasmic reticulum calcium homeostasis and cell death. Cell Calcium 69, 62–72. 10.1016/j.ceca.2017.05.003 28515000

[B98] Martinvalet D. (2018). The role of the mitochondria and the endoplasmic reticulum contact sites in the development of the immune responses. Cell Death Dis. 9, 336. 10.1038/s41419-017-0237-7 29491398 PMC5832423

[B99] Missiroli S. Bonora M. Patergnani S. Poletti F. Perrone M. Gafà R. (2016). PML at mitochondria-associated membranes is critical for the repression of autophagy and cancer development. Cell Rep. 16, 2415–2427. 10.1016/j.celrep.2016.07.082 27545895 PMC5011426

[B100] Miyao M. Cicalese S. Kawai T. Cooper H. A. Boyer M. J. Elliott K. J. (2020). Involvement of senescence and mitochondrial fission in endothelial cell pro-inflammatory phenotype induced by angiotensin II. Int. J. Mol. Sci. 21, 3112. 10.3390/ijms21093112 PMC7247685 32354103

[B101] Mo G. Liu X. Zhong Y. Mo J. Li Z. Li D. (2021). IP3R1 regulates Ca2+ transport and pyroptosis through the NLRP3/Caspase-1 pathway in myocardial ischemia/reperfusion injury. Cell Death Discov. 7, 31. 10.1038/s41420-021-00404-4 33568649 PMC7876122

[B102] Modesti L. Danese A. Vitto V. A. M. Ramaccini D. Aguiari G. Gafà R. (2021). Mitochondrial Ca2+ signaling in health, disease and therapy. Cells 10, 1317. 10.3390/cells10061317 34070562 PMC8230075

[B103] Moulis M. Grousset E. Faccini J. Richetin K. Thomas G. Vindis C. (2019). The multifunctional sorting protein PACS-2 controls mitophagosome formation in human vascular smooth muscle cells through mitochondria-ER contact sites. Cells 8, 638. 10.3390/cells8060638 PMC6627983 31242668

[B104] Munoz J. P. Ivanova S. Sánchez-Wandelmer J. Martínez-Cristóbal P. Noguera E. Sancho A. (2013). Mfn2 modulates the UPR and mitochondrial function via repression of PERK. EMBO J. 32, 2348–2361. 10.1038/emboj.2013.168 23921556 PMC3770335

[B105] Murley A. Nunnari J. (2016). The emerging network of mitochondria-organelle contacts. Mol. Cell 61, 648–653. 10.1016/j.molcel.2016.01.031 26942669 PMC5554544

[B106] Murphy S. E. Levine T. P. (2016). VAP, a versatile access point for the endoplasmic reticulum: Review and analysis of FFAT-like motifs in the VAPome. Biochim. Biophys. Acta 1861, 952–961. 10.1016/j.bbalip.2016.02.009 26898182

[B107] Myhill N. Lynes E. M. Nanji J. A. Blagoveshchenskaya A. D. Fei H. Simmen K. C. (2008). The subcellular distribution of calnexin is mediated by PACS-2. Mol. Biol. Cell 19, 2777–2788. 10.1091/mbc.e07-10-0995 18417615 PMC2441662

[B108] Naon D. Zaninello M. Giacomello M. Varanita T. Grespi F. Lakshminaranayan S. (2016). Critical reappraisal confirms that Mitofusin 2 is an endoplasmic reticulum–mitochondria tether. Proc. Natl. Acad. Sci. U. S. A. 113, 11249–11254. 10.1073/pnas.1606786113 27647893 PMC5056088

[B109] Nishimura A. L. Mitne-Neto M. Silva H. C. A. Richieri-Costa A. Middleton S. Cascio D. (2004). A mutation in the vesicle-trafficking protein VAPB causes late-onset spinal muscular atrophy and amyotrophic lateral sclerosis. Am. J. Hum. Genet. 75, 822–831. 10.1086/425287 15372378 PMC1182111

[B110] Oh S.-J. Lee J. K. Shin O. S. (2019). Aging and the immune system: The impact of immunosenescence on viral infection, immunity and vaccine immunogenicity. Immune Netw. 19, e37. 10.4110/in.2019.19.e37 31921467 PMC6943173

[B111] Papadopoulos C. Kirchner P. Bug M. Grum D. Koerver L. Schulze N. (2017). VCP/p97 cooperates with YOD1, UBXD1 and PLAA to drive clearance of ruptured lysosomes by autophagy. Embo J. 36, 135–150. 10.15252/embj.201695148 27753622 PMC5242375

[B112] Pavlova N. N. Thompson C. B. (2016). The emerging hallmarks of cancer metabolism. Cell Metab. 23, 27–47. 10.1016/j.cmet.2015.12.006 26771115 PMC4715268

[B113] Pence B. D. Yarbro J. R. (2018). Aging impairs mitochondrial respiratory capacity in classical monocytes. Exp. Gerontol. 108, 112–117. 10.1016/j.exger.2018.04.008 29655929

[B114] Pennanen C. Parra V. López-Crisosto C. Morales P. E. Campo A. D. Gutierrez T. (2014). Mitochondrial fission is required for cardiomyocyte hypertrophy mediated by a Ca2+-calcineurin signaling pathway. J. Cell Sci. 127, 2659–2671. 10.1242/jcs.139394 24777478 PMC4058110

[B115] Pernaute B. Pérez-Montero S. Nieto J. M. S. Gregorio A. D. Lima A. Lawlor K. (2022). DRP1 levels determine the apoptotic threshold during embryonic differentiation through a mitophagy-dependent mechanism. Dev. Cell 57, 1316–1330.e7. e7. 10.1016/j.devcel.2022.04.020 35597240 PMC9297746

[B116] Perocchi F. Gohil V. M. Girgis H. S. Bao X. R. McCombs J. E. Palmer A. E. (2010). MICU1 encodes a mitochondrial EF hand protein required for Ca2+ uptake. Nature 467, 291–296. 10.1038/nature09358 20693986 PMC2977980

[B117] Pietrangelo L. D’Incecco A. Ainbinder A. Michelucci A. Kern H. Dirksen R. T. (2015). Age-dependent uncoupling of mitochondria from Ca2⁺ release units in skeletal muscle. Oncotarget 6, 35358–35371. 10.18632/oncotarget.6139 26485763 PMC4742110

[B118] Pinton P. Giorgi C. Siviero R. Zecchini E. Rizzuto R. (2008). Calcium and apoptosis: ER-mitochondria Ca2+ transfer in the control of apoptosis. Oncogene 27, 6407–6418. 10.1038/onc.2008.308 18955969 PMC2844952

[B119] Poston C. N. Krishnan S. C. Bazemore-Walker C. R. (2013). In-depth proteomic analysis of mammalian mitochondria-associated membranes (MAM). J. Proteomics 79, 219–230. 10.1016/j.jprot.2012.12.018 23313214

[B120] Pulli I. Löf C. Blom T. Asghar M. Y. Lassila T. Bäck N. (2019). Sphingosine kinase 1 overexpression induces MFN2 fragmentation and alters mitochondrial matrix Ca2+ handling in HeLa cells. Biochim. Biophys. Acta. Mol. Cell Res. 1866, 1475–1486. 10.1016/j.bbamcr.2019.06.006 31220477

[B121] Puri R. Cheng X.-T. Lin M.-Y. Huang N. Sheng Z.-H. (2019). Mul1 restrains Parkin-mediated mitophagy in mature neurons by maintaining ER-mitochondrial contacts. Nat. Commun. 10, 3645. 10.1038/s41467-019-11636-5 31409786 PMC6692330

[B122] Rainbolt T. K. Saunders J. M. Wiseman R. L. (2014). Stress-responsive regulation of mitochondria through the ER unfolded protein response. Trends Endocrinol. Metab. 25, 528–537. 10.1016/j.tem.2014.06.007 25048297

[B123] Raturi A. Gutiérrez T. Ortiz-Sandoval C. Ruangkittisakul A. Herrera-Cruz M. S. Rockley J. P. (2016). TMX1 determines cancer cell metabolism as a thiol-based modulator of ER–mitochondria Ca2+ flux. J. Cell Biol. 214, 433–444. 10.1083/jcb.201512077 27502484 PMC4987292

[B124] Rebelo A. P. M. Bello F. D. Knedlik T. Kaar N. Volpin F. Shin S. H. (2020). Chemical modulation of mitochondria–endoplasmic reticulum contact sites. Cells 9, 1637. 10.3390/cells9071637 PMC7408517 32646031

[B125] Rezuchova I. Hudecova S. Soltysova A. Matuskova M. Durinikova E. Chovancova B. (2019). Type 3 inositol 1, 4, 5-trisphosphate receptor has antiapoptotic and proliferative role in cancer cells. Cell Death Dis. 10, 186. 10.1038/s41419-019-1433-4 30796197 PMC6385365

[B126] Rieusset J. (2018). The role of endoplasmic reticulum-mitochondria contact sites in the control of glucose homeostasis: An update. Cell Death Dis. 9, 388. 10.1038/s41419-018-0416-1 29523782 PMC5844895

[B127] Rohlenova K. Veys K. Miranda-Santos I. Bock K. D. Carmeliet P. (2018). Endothelial cell metabolism in health and disease. Trends Cell Biol. 28, 224–236. 10.1016/j.tcb.2017.10.010 29153487

[B128] Sano R. Annunziata I. Patterson A. Moshiach S. Gomero E. Opferman J. (2009). GM1-Ganglioside accumulation at the mitochondria-associated ER membranes links ER stress to Ca2+-dependent mitochondrial apoptosis. Mol. Cell 36, 500–511. 10.1016/j.molcel.2009.10.021 19917257 PMC2782904

[B129] Sassano M. L. Vliet A. R. V. Agostinis P. (2017). Mitochondria-associated membranes as networking platforms and regulators of cancer cell fate. Front. Oncol. 7, 174. 10.3389/fonc.2017.00174 28868254 PMC5563315

[B130] Sebastián D. Hernández-Alvarez M. I. Segalés J. Sorianello E. Munoz J. P. Sala D. (2012). Mitofusin 2 (Mfn2) links mitochondrial and endoplasmic reticulum function with insulin signaling and is essential for normal glucose homeostasis. Proc. Natl. Acad. Sci. U. S. A. 109, 5523–5528. 10.1073/pnas.1108220109 22427360 PMC3325712

[B131] Senga S. S. Grose R. P. (2021). Hallmarks of cancer—The new testament. Open Biol. 11, 200358. 10.1098/rsob.200358 33465324 PMC7881179

[B132] Sharma V. K. Ramesh V. Franzini-Armstrong C. Sheu S.-S. (2000). Transport of Ca2+ from sarcoplasmic reticulum to mitochondria in rat ventricular myocytes. J. Bioenerg. Biomembr. 32, 97–104. 10.1023/a:1005520714221 11768767

[B133] She H. Zhu Y. Deng H. Kuang L. Fang H. Zhang Z. (2021). Protective effects of dexmedetomidine on the vascular endothelial barrier function by inhibiting mitochondrial fission via ER/mitochondria contact. Front. Cell Dev. Biol. 9, 636327. 10.3389/fcell.2021.636327 33777946 PMC7991806

[B134] Shi J. Yang Y. Cheng A. Xu G. He F. (2020). Metabolism of vascular smooth muscle cells in vascular diseases. Am. J. Physiol. Heart Circ. Physiol. 319, H613–H631. 10.1152/ajpheart.00220.2020 32762559

[B135] Shirokova O. M. Pchelin P. V. Mukhina I. V. (2020). MERCs. The novel assistant to neurotransmission? Front. Neurosci. 14, 589319. 10.3389/fnins.2020.589319 33240039 PMC7680918

[B136] Sikora E. Bielak-Zmijewska A. Dudkowska M. Krzystyniak A. Mosieniak G. Wesierska M. (2021). Cellular senescence in brain aging. Front. Aging Neurosci. 13, 646924. 10.3389/fnagi.2021.646924 33732142 PMC7959760

[B137] Silva B. S. C. DiGiovanni L. Kumar R. Carmichael R. E. Kim P. K. Schrader M. (2020). Maintaining social contacts: The physiological relevance of organelle interactions. Biochim. Biophys. Acta. Mol. Cell Res. 1867, 118800. 10.1016/j.bbamcr.2020.118800 32712071 PMC7377706

[B138] Simmen T. Aslan J. E. Blagoveshchenskaya A. D. Thomas L. Wan L. Xiang Y. (2005). PACS-2 controls endoplasmic reticulum-mitochondria communication and Bid-mediated apoptosis. EMBO J. 24, 717–729. 10.1038/sj.emboj.7600559 15692567 PMC549619

[B139] Simmen T. Herrera-Cruz M. S. (2018). Plastic mitochondria-endoplasmic reticulum (ER) contacts use chaperones and tethers to mould their structure and signaling. Curr. Opin. Cell Biol. 53, 61–69. 10.1016/j.ceb.2018.04.014 29870872

[B140] Simón L. Campos A. Leyton L. Quest A. F. G. (2020). Caveolin-1 function at the plasma membrane and in intracellular compartments in cancer. Cancer Metastasis Rev. 39, 435–453. 10.1007/s10555-020-09890-x 32458269 PMC7311495

[B141] Song B.-L. Sever N. DeBose-Boyd R. A. (2005). Gp78, a membrane-anchored ubiquitin ligase, associates with insig-1 and couples sterol-regulated ubiquitination to degradation of HMG CoA reductase. Mol. Cell 19, 829–840. 10.1016/j.molcel.2005.08.009 16168377

[B142] Spazzafumo L. Mensà E. Matacchione G. Galeazzi T. Zampini L. Recchioni R. (2017). Age-related modulation of plasmatic beta-Galactosidase activity in healthy subjects and in patients affected by T2DM. Oncotarget 8, 93338–93348. 10.18632/oncotarget.21848 29212153 PMC5706799

[B143] Spurlock B. Tullet J. Hartman J. L. Mitra K. (2020). Interplay of mitochondrial fission-fusion with cell cycle regulation: Possible impacts on stem cell and organismal aging. Exp. Gerontol. 135, 110919. 10.1016/j.exger.2020.110919 32220593 PMC7808294

[B144] Srivastava S. (2017). The mitochondrial basis of aging and age-related disorders. Genes 8, 398. 10.3390/genes8120398 PMC5748716 29257072

[B145] Szabadkai G. Bianchi K. Várnai P. Stefani D. D. Wieckowski M. R. Cavagna D. (2006). Chaperone-mediated coupling of endoplasmic reticulum and mitochondrial Ca2+ channels. J. Cell Biol. 175, 901–911. 10.1083/jcb.200608073 17178908 PMC2064700

[B146] Tang B. L. (2015). MIRO GTPases in mitochondrial transport, homeostasis and pathology. Cells 5, 1. 10.3390/cells5010001 PMC4810086 26729171

[B147] Tang X. Li P.-H. Chen H.-Z. (2020). Cardiomyocyte senescence and cellular communications within myocardial microenvironments. Front. Endocrinol. 11, 280. 10.3389/fendo.2020.00280 PMC7253644 32508749

[B148] Tubbs E. Chanon S. Robert M. Bendridi N. Bidaux G. Chauvin M.-A. (2018). Disruption of mitochondria-associated endoplasmic reticulum membrane (MAM) integrity contributes to muscle insulin resistance in mice and humans. Diabetes 67, 636–650. 10.2337/db17-0316 29326365

[B149] Tubbs E. Theurey P. Vial G. Bendridi N. Bravard A. Chauvin M.-A. (2014). Mitochondria-associated endoplasmic reticulum membrane (MAM) integrity is required for insulin signaling and is implicated in hepatic insulin resistance. Diabetes 63, 3279–3294. 10.2337/db13-1751 24947355

[B150] Ueasilamongkol P. Khamphaya T. Guerra M. T. Rodrigues M. A. Gomes D. A. Kong Y. (2020). Type 3 inositol 1, 4, 5‐trisphosphate receptor is increased and enhances malignant properties in cholangiocarcinoma. Hepatology 71, 583–599. 10.1002/hep.30839 31251815 PMC6934938

[B151] Umemoto T. Hashimoto M. Matsumura T. Nakamura-Ishizu A. Suda T. (2018). Ca2+–mitochondria axis drives cell division in hematopoietic stem cells. J. Exp. Med. 215, 2097–2113. 10.1084/jem.20180421 29946000 PMC6080917

[B152] Vance J. E. (2014). MAM (mitochondria-associated membranes) in mammalian cells: Lipids and beyond. Biochim. Biophys. Acta 1841, 595–609. 10.1016/j.bbalip.2013.11.014 24316057

[B153] Verfaillie T. Rubio N. Garg A. D. Bultynck G. Rizzuto R. Decuypere J.-P. (2012). PERK is required at the ER-mitochondrial contact sites to convey apoptosis after ROS-based ER stress. Cell Death Differ. 19, 1880–1891. 10.1038/cdd.2012.74 22705852 PMC3469056

[B154] Vilas J. M. Carneiro C. Silva‐Álvarez S. D. Ferreirós A. González P. Gómez M. (2018). Adult Sox2+ stem cell exhaustion in mice results in cellular senescence and premature aging. Aging Cell 17, e12834. 10.1111/acel.12834 30129215 PMC6156495

[B155] Völgyi K. Badics K. Sialana F. J. Gulyássy P. Udvari E. B. Kis V. (2018). Early presymptomatic changes in the proteome of mitochondria-associated membrane in the APP/PS1 mouse model of alzheimer’s disease. Mol. Neurobiol. 55, 7839–7857. 10.1007/s12035-018-0955-6 29468564

[B156] Wang C. Dai X. Wu S. Xu W. Song P. Huang K. (2021). FUNDC1-dependent mitochondria-associated endoplasmic reticulum membranes are involved in angiogenesis and neoangiogenesis. Nat. Commun. 12, 2616. 10.1038/s41467-021-22771-3 33972548 PMC8110587

[B157] Wang P. T. C. Garcin P. O. Fu M. Masoudi M. St-Pierre P. Panté N. (2015a). Distinct mechanisms controlling rough and smooth endoplasmic reticulum contacts with mitochondria. J. Cell Sci. 128, 2759–2765. 10.1242/jcs.171132 26065430

[B158] Wang W. Xie Q. Zhou X. Yao J. Zhu X. Huang P. (2015b). Mitofusin-2 triggers mitochondria Ca2+ influx from the endoplasmic reticulum to induce apoptosis in hepatocellular carcinoma cells. Cancer Lett. 358, 47–58. 10.1016/j.canlet.2014.12.025 25541060

[B159] Wang Y.-J. Huang J. Liu W. Kou X. Tang H. Wang H. (2017). IP3R-mediated Ca2+ signals govern hematopoietic and cardiac divergence of Flk1+ cells via the calcineurin–NFATc3–Etv2 pathway. J. Mol. Cell Biol. 9, 274–288. 10.1093/jmcb/mjx014 28419336

[B160] Wiel C. Lallet-Daher H. Gitenay D. Gras B. Calvé B. L. Augert A. (2014). Endoplasmic reticulum calcium release through ITPR2 channels leads to mitochondrial calcium accumulation and senescence. Nat. Commun. 5, 3792. 10.1038/ncomms4792 24797322

[B161] Wiley C. D. Sharma R. Davis S. S. Lopez-Dominguez J. A. Mitchell K. P. Wiley S. (2021). Oxylipin biosynthesis reinforces cellular senescence and allows detection of senolysis. Cell Metab. 33, 1124–1136.e5. e5. 10.1016/j.cmet.2021.03.008 33811820 PMC8501892

[B162] Wójcik C. Rowicka M. Kudlicki A. Nowis D. McConnell E. Kujawa M. (2006). Valosin-containing protein (p97) is a regulator of endoplasmic reticulum stress and of the degradation of N-end rule and ubiquitin-fusion degradation pathway substrates in mammalian cells. Mol. Biol. Cell 17, 4606–4618. 10.1091/mbc.e06-05-0432 16914519 PMC1635394

[B163] Xie J.-H. Li Y.-Y. Jin J. (2020). The essential functions of mitochondrial dynamics in immune cells. Cell. Mol. Immunol. 17, 712–721. 10.1038/s41423-020-0480-1 32523116 PMC7331746

[B164] Xu H. Guan N. Ren Y.-L. Wei Q.-J. Tao Y.-H. Yang G.-S. (2018). IP3R-Grp75-VDAC1-MCU calcium regulation axis antagonists protect podocytes from apoptosis and decrease proteinuria in an Adriamycin nephropathy rat model. BMC Nephrol. 19, 140. 10.1186/s12882-018-0940-3 29907098 PMC6003198

[B165] Xu S. Peng G. Wang Y. Fang S. Karbowski M. (2011). The AAA-ATPase p97 is essential for outer mitochondrial membrane protein turnover. Mol. Biol. Cell 22, 291–300. 10.1091/mbc.e10-09-0748 21118995 PMC3031461

[B166] Yang P.-C. Jafri M. S. (2020). Ca2+ signaling in T lymphocytes: The interplay of the endoplasmic reticulum, mitochondria, membrane potential, and CRAC channels on transcription factor activation. Heliyon 6, e03526. 10.1016/j.heliyon.2020.e03526 32181396 PMC7063158

[B167] Yoboue E. D. Sitia R. Simmen T. (2018). Redox crosstalk at endoplasmic reticulum (ER) membrane contact sites (MCS) uses toxic waste to deliver messages. Cell Death Dis. 9, 331. 10.1038/s41419-017-0033-4 29491367 PMC5832433

[B168] Zakrzewski W. Dobrzyński M. Szymonowicz M. Rybak Z. (2019). Stem cells: Past, present, and future. Stem Cell Res. Ther. 10, 68. 10.1186/s13287-019-1165-5 30808416 PMC6390367

[B169] Zhang J. Zhang F. Wang Y. (2021a). Mitofusin-2 enhances mitochondrial contact with the endoplasmic reticulum and promotes diabetic cardiomyopathy. Front. Physiol. 12, 707634. 10.3389/fphys.2021.707634 34305656 PMC8298037

[B170] Zhang S.-S. Zhou S. Crowley-McHattan Z. J. Wang R.-Y. Li J.-P. (2021b). A review of the role of endo/sarcoplasmic reticulum-mitochondria Ca2+ transport in diseases and skeletal muscle function. Int. J. Environ. Res. Public Health 18, 3874. 10.3390/ijerph18083874 33917091 PMC8067840

[B171] Zhang X. Gibhardt C. S. Will T. Stanisz H. Körbel C. Mitkovski M. (2019). Redox signals at the ER–mitochondria interface control melanoma progression. Embo J. 38, e100871. 10.15252/embj.2018100871 31304984 PMC6669928

[B172] Zheng M. Kim S. Joe Y. Back S. H. Cho H. R. Kim H. P. (2012). Sensing endoplasmic reticulum stress by protein kinase RNA‐like endoplasmic reticulum kinase promotes adaptive mitochondrial DNA biogenesis and cell survival via heme oxygenase‐1/carbon monoxide activity. Faseb J. 26, 2558–2568. 10.1096/fj.11-199604 22391129

[B173] Zhou H. Yue Y. Wang J. Ma Q. Chen Y. (2018). Melatonin therapy for diabetic cardiomyopathy: A mechanism involving syk-mitochondrial complex I-SERCA pathway. Cell. Signal. 47, 88–100. 10.1016/j.cellsig.2018.03.012 29601906

[B174] Ziegler D. V. Martin N. Bernard D. (2021a). Cellular senescence links mitochondria-ER contacts and aging. Commun. Biol. 4, 1323. 10.1038/s42003-021-02840-5 34819602 PMC8613202

[B175] Ziegler D. V. Vindrieux D. Goehrig D. Jaber S. Collin G. Griveau A. (2021b). Calcium channel ITPR2 and mitochondria–ER contacts promote cellular senescence and aging. Nat. Commun. 12, 720. 10.1038/s41467-021-20993-z 33526781 PMC7851384

